# Hilbert’s problems, Kant, and decidability

**DOI:** 10.1007/s00407-025-00350-y

**Published:** 2025-08-11

**Authors:** Moritz Bodner

**Affiliations:** Universität Wien, Vienna, Austria

## Abstract

I show on the basis of unpublished sources how Hilbert’s conviction of the solvability of all mathematical problems originated from an engagement with Kant’s philosophy of mathematics. Furthermore, I consider other sense of the “solvability” or “decidability” of mathematical problems which Hilbert thought about later: decidability in finitely many steps, which is an issue Hilbert inherited from Kronecker, “finitistic decidability” which Hilbert develops by reflecting on Kronecker’s methodological strictures, and finally the decision-problem as raised by Behmann in the 1920s. I argue that these different preoccupations have different historical and biographical roots, and should also be kept conceptually distinct.

## Preamble

Even amongst people who have no (clear) idea of David Hilbert as a historical figure nor detailed knowledge of his mathematical work “Hilbert’s Problems” are famous; or at least the idea—and the allure—of famously unresolved problems of pure mathematics is.^1^ Hilbert presented his selection of problems “from whose discussion one can expect a furtherance of the science [of mathematics]”^2^ at the World Congress of Mathematics held in Paris in the year 1900.^3^ But Hilbert’s thinking about mathematical problems in general, and his effort to fixate particular problems whose investigation would be especially fruitful, extends beyond his lecture in 1900, both in scope and in time.

In this article, I aim to survey the aspects of Hilbert’s thinking that bear on mathematics from a generalist, broadly speaking: “methodologico-programmatic” point of view. I will focus, more specifically, on those strands of Hilbert’s thinking which become, in one way or another, entangled with the question of the “solvability” or “decidabilty” (in sense to be clarified in due course) of mathematical problems. In particular, I will discuss, first (in Sect. 1), the conviction Hilbert famously announced to the World Congress of Mathematics in 1900: that all (properly formulated) mathematical problems are solvable, and its historical, biographical and philosophical backdrop. Then (in Sect. 2) I will discuss how the lines of thought leading Hilbert to this conviction continue to develop after 1900. One issue Hilbert keeps coming back to in this connection is the question of whether mathematical problems can not only be solved « in principle »but by means of finitely many steps of calculation or proof. This concern with the finitude of the processes leading to the resolution of mathematical problems Hilbert appears to have inherited from Kronecker (as I discuss in Sect. 3.1), but Hilbert also develops it further in a certain direction which leads him to one central stricture later enshrined in what he calls the “finitist standpoint”. Finally (in Sect. 3.2) I discuss the relation of these various senses of “solvablility” or “decidability” that preoccupy Hilbert with the sense relevant to the so-called “Decision Problem” as raised by Heinrich Behmann in 1922. In conclusion, I offer an overview of the different sense of “decidability” which are, as I will have shown, of importance to Hilbert at different phases in his career for different reasons.

## Hilbert’s 24th problem

The full extent of Hilbert’s thinking about methodological issues is revealed really only by writings which remain unpublished and are held as manuscripts amongst Hilbert’s papers by the Staats- und Universitätsbibliothek (SUB) in Göttingen,^4^ especially by three notebooks Hilbert kept to record all kinds of thoughts and ideas between the years 1885^5^ and 1912^6^ or so. In the third of these notebooks^7^ one finds a draft for what Hilbert seems to have at some point considered including in his famous list of Mathematical Problems as the final, $24\text {th}$ entry^8^:

As the 24th Problem in my Paris address, I wanted to pose the question: criteria for the simplicity, or rather to give a proof of the greatest simplicity of certain proofs. In general, to develop a theory of methods of proof in mathematics. There can under given conditions only be one simplest proof. Generally, whenever one has two proofs for a proposition one must not rest until one has reduced each to the other or recognised precisely which presuppositions (or aides) have been used in these proofs: When there are 2 paths, one must not just walk these paths or search new ones, but chart the entire region which lies between them. [...^9^] Since every process of adding [is] an application of the commutative law of addition etc. – which always corresponds to geometric propositions or logical inferences, one can count those and so decide, for example, when proving certain propositions of elementary geometry (Pythagoras, or about the special points in a triangle) very well which proof is the simplest.^10^

This entry marks the earliest appearance in Hilbert’s writings of an idea Hilbert later championed: developing a theory of mathematical proof as distinctive mathematical discipline^11^; though the specific issues touched upon here—singling out a simplest proof for a given result^12^ and comprehensively surveying all alternative proofs—did not figure prominently in the proof-theory Hilbert eventually developed, starting in 1922.^13^ But more interesting than this entry’s « prophetic quality »is the origin and afterlife of these considerations in their own time. They can be traced through the volumes of Hilbert’s notebooks and turn out to be tied to other strands of reflection which led Hilbert to think, and finally to speak at the World Congress, about mathematical problems in general; that may be why Hilbert saw this as the natural last entry for his list of problems.

This article is dedicated to narrating that history. It is meant also to highlight the richness of Hilbert’s notebooks as still largely untapped sources recording one outstanding mathematician’s reflections on mathematics, and mathematicians, both of his time and in general terms. Finally, the lines of reflection which run to Hilbert’s 1900 address also bring into focus how thinking about mathematics intermingled in Hilbert’s mind with a serious engagement with philosophy.

Along the way, I will also highlight other aspects, bits and pieces of Hilbert’s unpublished writings not yet widely known within the mathematical or philosophical community.

As a point of departure I take Hilbert’s earliest reflections on the nature of mathematics, traces of which can still be recognised in the published text of Hilbert’s 1900 address. From there I shall work myself forward in time along the lines of development running through Hilbert’s notes which finally issue in Hilbert’s sketch for a $24\text {th}$ entry for his famous list of Mathematical Problems.

### Hilbert and Kant’s theory of mathematics

Speaking with a sweeping generality about mathematical problems and mathematics as a whole in the opening remarks of his Paris address, Hilbert famously expressed his conviction

which every mathematicians certainly shares but nobody has, at least thus far, supported by proofs [...] that any determinate mathematical problem must necessarily admit of a rigorous treatment, be it that one succeeds in giving an answer to the question posed, be it that the impossibility of its solution and thus the necessity of the failure of all attempts is demonstrated.^14^

Although Hilbert goes on to refer to it as an “axiom” (“of the solvability of every problem”), this conviction is not simply an article of faith for Hilbert. Even in the text of his address Hilbert briefly cites recent mathematical advances, such as the proof of the transcendence of $\pi$ which resolved the age-old problem of squaring the circle by showing “the impossibility of its solution”,^15^ as grounds for his conviction that every mathematical problem, if only it has been clearly formulated, can be resolved one way or another: positively by proving it as a theorem or negatively by what Hilbert eventually comes to call an “impossibility-proof”.^16^ Hilbert also gestures towards “other philosophical reasons” supporting it, without indicating in any detail what he has in mind. Hilbert’s unpublished notebooks can help spell out this allusion. They show that Hilbert had been thinking about the matter since the late 1880s and how he arrived at his conviction step by step.^17^

Early on in the first notebook, one finds the following entry:

Assuming that there were no practical difficulties, is the human mind capable of solving all questions it poses for itself? Must it be possible to come to a decision about the quadrature of the circle, is there a well-defined question referring to things given to the senses which must have an answer and yet cannot be answered. Can one give a general decisive proof that all knowledge must be possible?^18^

The quest for a “proof that all knowledge must be possible” expressed at the end of this passage can be seen as echoed, if distantly, in Hilbert’s assertion in 1900 that, although everybody “certainly shares” the conviction that all properly formulated problems can be resolved, “nobody has [...] supported [it] by proofs”. An entry in the third notebook (on p. 104, likely dating from the early to mid-1900s) reveals that Hilbert came to believe that his foundational work, which he there refers to summarily as “my theory of logic-arithmetic”,^19^ would enable him to give such a proof “that there is no ignorabimus in mathematics”^20^ whose absence he had lamented in 1900.^21^

Turn back to the entry I quoted from the first notebook, which I estimate dates from around 1890.^22^ The terminology Hilbert uses in this entry is unmistakably philosophical: The last question to be formulated there concerns the possibility of “knowledge” (“Erkenntnis”); and Hilbert began by asking whether there can be a question concerned with “things given to the senses” (“sinnliche Dinge”), which the human mind (“der menschliche Verstand”) may be incapable of answering.

The questions Hilbert raises in this notebook-entry—concerning the possibility of knowledge and the limits of the human mind’s reach—are reminiscent of those which Immanuel Kant posed as the leading questions of his “critical” philosophy. Indeed, one can, I think, explain quite well why Hilbert raises, in particular, the case of “things given to the senses” as potentially most problematic if one views this discussion as situated before the backdrop of Kant’s theory of knowledge. As a matter of fact, there is a good reason, beyond the philosophical roots of the terminology and the affinity of Hilbert’s questions to Kant’s, to think that this entry testifies to Hilbert’s engagement with Kant’s philosophical position: A few pages earlier, one finds entries which mention Kant’s name explicitly. In one of them Hilbert comes to the conclusion that “Kant is right [about numbers].”^23^

Then, in the entry immediately following this definitive assertion, Hilbert writes:

In all other sciences [there is] matter, of which one knows not whence it comes, one knows only that it is there. Here is pure thought, pure philosophy. Kant was the first to feel this, but did not prove it; he has begun something, but not finished it.^24^

Although Hilbert does not explicitly specify how the local metaphor is to be spelled out—i.e. what scientific discipline is supposed to be “here”—, it stands to reason that Hilbert is talking about mathematics; which is, indeed, the only (theoretical) discipline within Kant’s theory of the sciences not to presuppose any “matter” (i.e., according to Kant’s theory, anything given to the senses).

Kant articulates his philosophical views of mathematics on two occasions in the second half of the *Critique of Pure Reason*, namely: in the section on the so-called “Antinomy of Pure Reason” – the longest sub-section of the so called “Transcendental Dialectics”^25^ – and in the “Transcendental Doctrine of Method”, where Kant calls “mathematics a shining example of pure reason extending itself by its own means without the aid of experience”.^26^

This singular, special character of mathematics as a “pure science of reason” is what Kant emphasises also in sub-section 4 of the section on the “Antinomy of Pure Reason”, where Kant clarifies that from his point of view pure mathematics is the only such science within the theoretical domain.^27^ The hallmark of what Kant calls “pure sciences of reason”, as Kant characterises them in this sub-section, consists precisely in the feature Hilbert seizes on,^28^ namely, that

their nature brings with it that every question which occurs in the[se sciences] must without fail be capable of being answered without any doubt remaining based on what knowledge one has because the answer arises from the same sources as the question so that it is perfectly impermissible to claim inescapable ignorance; rather, resolution can be demanded.^29^

It is, according to Kant’s philosophy of mathematics, *because* mathematics does not rely on external matter given to the senses – so that all its content, problems as much as answers, originate accordingly in the human mind alone –, *that it is capable of solving any problem* which can be posed within its domain, and for this same reason mathematicians are – as Hilbert stressed in 1900 (see below) and Kant himself had already stressed (see above) – *compelled* to actually do so.

### Hilbert’s rejection of irremediable ignorance

This summary paraphrase of Kant’s view of mathematics bears a striking resemblance to Hilbert’s perhaps most famous utterance, his dictum that “in mathematics there is no ignorabimus”:

This conviction of the solvability of any mathematical problem is a strong motivation whilst at work; we hear within ourselves the constant call: *There is the problem, seek its solution. You can find it by means of pure thought; because in mathematics there is no Ignorabimus!*^30^

In light of Hilbert’s engagement with Kant, as documented in Hilbert’s first notebook (and with Kant’s views on mathematics, in particular, as documented by the passage quoted in footnote 23), this passage seems like little more than an exceedingly compressed summary and affirmation of Kant’s views on mathematics, as Hilbert had come to understand and adopted them for himself^31^: For Hilbert mathematics is a pure theoretical science in Kant’s sense, that is to say, it relies on “pure thought” both to pose its problems as well as to subsequently find solutions for them.

Other entries in Hilbert’s notebooks suggest that Hilbert became convinced that “in mathematics there is no ignorabimus” *as a result of reading Kant*. One finds the process documented by the entries in the first of Hilbert’s notebooks beginning with the entry about Kant I quoted (on p. 7, footnote 24), followed by entries leading eventually to an expression of Hilbert’s conviction that every mathematical problem can be resolved and that there can therefore be no irremediable ignorance. Indeed, Hilbert’s dictum already appears fully formulated in the first volume of Hilbert’s notebooks – so, even in light of the uncertainty inherent in dating these entries, « a good decade »(see footnote 22) before Hilbert announced it to the world in Paris in 1900 – on p. 72:

Mathematics is the only science for which there is no ignorabimus but rather we must say with the greatest comprehensiveness and with respect to all problems, however difficult: *noscemus*.^32^

That Hilbert looked back at these notes, written ten or more years earlier, when preparing his address in 1900 derives some support from the following observation. On the same page as the entries I discussed on page 7, there is an entry consisting originally only of a single phrase:

On a fundamental axiom of the human mind.^33^

However, Hilbert returned to and revised this entry – likely later –, adding to it – in markedly different (much smaller and less well-mannered) handwriting^34^ – a suggestion for what this “fundamental axiom”, which the original entry left unspecified, might be:

Maybe that every problem is solvable.? [sic]

Which is what Hilbert claims in 1900 (see page 4).

That the key-word “ignorabimus” (“We will not know.”), and Hilbert’s contrary “noscemus” (“We will know.”), appears only relatively late in the first notebook and seemingly only to furnish new guises for ideas Hilbert already held and expressed previously, suggests that Hilbert came to hold the conviction that all properly formulated mathematical problems can be solved (positively or negatively) originally as a result of his engagement with Kant’s philosophy and that Hilbert learned only subsequently of du Bois-Raymond’s “Ignoramus et ignorabimus!”-dictum, to which he then explicitly opposed also in formulation an idea he had already accepted independently before.^35^

I don’t mean to call into question that Hilbert *eventually* chose and subsequently stuck to his “No ignorabimus!”-formulation to embed his claim in the controversy that followed du Bois-Reymond’s lecture,^36^ only that du Bois-Reymond’s assertion *caused* Hilbert to think about the issue and that Hilbert took the contrary position he took *initially* in opposition to du Bois-Reymond; rather, it seems to me, judging by the notebook-entries I have surveyed, that Hilbert thought and made up his mind about the issue independently, on the basis of reading Kant and appropriating his doctrines.

The « missing link »between the entries concerned with Kant and the entry where Hilbert announces his “Noscemus!”-slogan, at which point, like in Hilbert’s 1900 address to the mathematical congress, Kant is no longer mentioned, is a lengthy entry on p. 37 of the first notebook, where Hilbert discusses the general form shared by all mathematical problems, or so he claims:

Every mathematical problem can be reduced to the following question: A law is given, by means of which to any given non-terminating sequence consisting only of zero and 1 [...] a certain other such sequence [...] can be constructed (by means of calculatory operations: seek the greatest whole, a divisor etc. No playing dice...). One must decide by a finite number of operations whether in any sequence a 0 occur or whether all sequences consist of ones only?

I claim: Any such decision is possible by way of a finite number of operations (calculatory operations). That is: There is no law with respect to which the decision would not be possible by way of a finite number of operations. That is: Every mathematical problem is solvable. Anything the human mind can reach (by means of pure thinking without matter) can also be resolved. There is only one problem.^37^ (e.g. quadrature of the circle. Does $\pi =3.14\dots$ have 10 immediately adjacent 7s etc.)

The assumption of the possibility is taken for granted from the outset.^38^

One still finds here the philosophical terminology used in the entries documenting Hilbert’s engagement with Kant (as well as, again, Hilbert’s, and Kant’s, default example of a problem whose solution is to be expected: the quadrature of the circle), but also already the formulation of Hilbert’s conviction one finds in the later entry I quoted – that all problems are solvable – and even the compressed (Kantianising) rationale adduced (in even more compressed form) in the 1900 address, namely, that the solvability of every mathematical problem is owed to the fact that mathematics is based on “pure thinking without matter” (as Hilbert puts it here, which in 1900 becomes “finding [solutions to problems] by means of pure thought” (see p. 9, footnote 30)).^39^

The various entries I have quoted lead, it seems to me, almost continuously from Hilbert study and appropriation of Kant’s position that mathematics is a “pure science of reason” to Hilbert’s dictum in the 1900 address that all mathematical problems, if they are properly formulated, admit of a (positive or negative) solution, and *clarifies* the latter by shedding light on Hilbert’s (philosophical) rationale, on *why* he takes every mathematical problem to be solvable; I contend that something like these considerations are, in any case, the background of Hilbert’s allusion in his 1900 address to “other philosophical considerations” supporting the conviction that every mathematical problem is solvable. As these passages reveal, Hilbert’s “axiom of the solvability of all problems” is only improperly so-called: It is not a naively held article of faith for Hilbert, but rather a consequence of Hilbert’s acceptance of more fundamental philosophical assumptions about the nature of mathematics, which Hilbert came to accept relatively early on in his intellectual development^40^ as part of a broadly Kantian theory of science in general, and of mathematics specifically^41^: Because mathematical knowledge does not depend on any “matter”, all relevant tools for solving mathematical problems reside in what Hilbert calls “pure thinking”, so they are always at hand and a solution to any mathematical problem, or at least its possibility, along with them.^42^

Hilbert’s conception of mathematics as a “science of pure thought” casts Hilbert in the role of what philosophers might call “an objective idealist” with respect to (pure) mathematics,^43^ i.e. somebody who believed that, fundamentally, mathematics was a product of human cognitive activity – this is the “idealist” element: ideas are prior to reality –, but also that the cognitive capacities enabling human cognition were universally shared and the same for all human beings – this is the “objective” element: mathematical reality, as constituted by human cognition, is as it is independently of any particular individual’s beliefs about it – (whilst, for instance, Brouwer may have believed, to the contrary, that such universality of cognitive capacities cannot be presupposed.^44^

## The (methodo-)logical analysis of mathematics

Coming to accept in this way – around 1890 or so – that every mathematical problem can be solved was for Hilbert only the beginning of a process of reflection on the nature, structure and order of (mathematical) problems generally. Further and related questions would continue to preoccupy him with varying intensity for years to come. Once again, the notebooks offer ample testimony of this preoccupation. The entries they contain show how, after first broaching the topic he subsequently frames as a potential $24\text {th}$ Problem, Hilbert raised and explored also other, related methodological questions and issues about mathematics generally.

I will now highlight some of the pertinent entries, beginning with one in particular which provides a link between the conviction that all mathematical problems are solvable and the issue about (comparative) simplicity Hilbert thought to (but did not) pose as his $24\text {th}$ Problem.

### Towards the $24\text {th}$ problem

The first entry recording Hilbert’s thinking about mathematical problems generally is found also in the first notebook on pp. 52f., some time after the entries documenting Hilbert’s engagement with Kant (mainly p. 28ff. of the first notebook) and the entry where Hilbert first formulated his later famous dictum (on p. 37 of the first notebook). In a unusually long entry – entries in the first notebook are usually not longer than a few lines –, Hilbert, so to speak, picks up where his engagement with Kant left him, i.e. having accepted the solvability of every problem:

Mathematics has the advantage over other sciences that one can definitively say whether a question is dealt with. [...] For mathematics the danger lies in arbitrariness getting ahead of itself – corresponding to the fact that it is the mind by itself which sets the task, solves it and critically evaluates and esteems it according to its own taste and without external standards: Strive for large striking aspects and the prescription one imposes onto oneself as a duty, to view and compare every result with respect to the science as a whole. This yields a better way of working through the available stuff than if everyone just keeps digging through their own trash, which nobody else cares about.^45^ [...] Not dealing with particular questions is what matters (that is only an overarching motivation, a looming goal, exciting bait) but that the proud building of science rises before our mind’s eye harmoniously and unified, as from a single block of stone.^46^ A question *Q*, for which the next superordinate question $Q^2$ has been answered with yes, is to be esteemed less highly – perhaps a criterion for the importance of the question. In any case, there is a great difference even where the next superordinate question $Q^2$ has been answered, whether I show only that something is possible by a finite number of processes (as with my proof for the finitude of the invariants of ternary forms) or whether I really state an upper limit for the number of processes, so that it is only a question of time and investing labour. Furthermore there is still the question whether there are problems in mathematics which cannot be dealt with in a predeterminately small amount of time? etc.^47^

Following a rather compressed summary of the Kantian rationale for the solvability of all problems – “the mind by itself sets the task, solves it [...] without external standards” –, all kinds of different, broadly speaking: “methodological” questions about mathematical problems are raised here in no discernible order and without a clean separation between them.

Included in this jumble are kernels of the questions Hilbert’s $24\text {th}$ Problem clusters around: There is, first of all, the idea “to view and compare every result with respect to the science as a whole”, which, duly modified to apply to proofs, appears also in the sketch of Hilbert’s $24\text {th}$ Problem as the maxim to “not just walk [one of two] paths or search new ones, but chart the entire region”.

A second connection between this entry and Hilbert’s sketch for a $24\text {th}$ Problem runs through the quantification of steps of calculation, implied here in Hilbert’s talk about the “number of processes” needed to solve a problem as in the suggestion, in Hilbert’s sketch for his $24\text {th}$ Problem that one can “count” operations in order to determine the relative simplicity of a given proof.

Of that main preoccupation central to Hilbert’s sketch for a $24\text {th}$ Problem – comparing alternatives proofs with respect, specifically, to their simplicity – no trace is apparent in this entry, however. I believe that this omission is tolerably insubstantial, upon reflection: In his sketch, Hilbert, after all, says only that one can compare proofs with respect to their simplicity *because* one can count the operations comprised in each, so relative simplicity, as Hilbert there conceives it, appears to be merely a matter of one proof involving *a greater number* or, respectively, fewer operations than another—an rather rudimentary conception of relative simplicity.

This criterion, so far as it goes, provides no obvious guideline for what would arguably be some of the most interesting applications, e.g. how to compare with respect to their relative simplicity proofs establishing the same result « by different methods »(e.g. to use an example close to Hilbert’s own heart around 1900: Are the proofs one can give of geometric theorems in analytic geometry in the relevant sense “simpler” than the proofs of the same theorems given in synthetic/axiomatic geometry, or conversely; and, either way: what makes it so?); or which to prefer when two proofs involve the same total number of operations but either different specific operations or the same operations but in different numbers or merely arranged in a different order.^48^

### The logical analysis of mathematics

If the criterion for simplicity Hilbert suggests in his sketch stands any chance of being widely applicable, also to such interesting cases, Hilbert must, it seems, be committed to presupposing an assumption to the effect that all proofs can ultimately be analysed in terms of the same handful of fundamental cognitive operations,^49^ so that merely considering the number of operations needed to obtain the result is a sufficient criterion for comparing the relative simplicity of any two alternative proofs (and even this is still a charitable interpretation of Hilbert’s criterion, insofar as it ignores cases, which seem perfectly possible in principle, of proofs with the same total number of operations, differing only in how often specific operations are used instead of others, or in which order the same number of the same operations are deployed, which may certainly make a big psychological difference). Although Hilbert, as far as I can see, never comes close to addressing this difficulty, or to even formulate it clearly and explicitly, he seems to have been aware that something further is needed, as the following entry found at the beginning of the second notebook shows:

Formulate a theory of calculation, i.e. investigate what the advantages of familiar arithmetical formulas and sentences (...) for calculating certain problems consist in and how one can estimate these advantages [themselves] by means of calculation. (Maybe by introducing certain elementary calculatory operations (addition, multiplication, exponentiation) as units and determine the number of units to be applied in different paths one treads in calculating. How many calculatory units, for example, are required to determine the so-and-so-manieth position in the decimal expansion of $\pi$? What is the quickest way of calculating *n*!? [...]^50^

Whilst this entry still focuses on examples obtained by actual calculation and makes little headway towards quantifying the « cognitive work »involved in actual proofs – conceptual argumentation as opposed to numerical calculations^51^ – and whilst, as far as I am aware, such programmatic statements as this entry are as close as Hilbert ever got to actually developing a “theory of calculation”, this passage can serve as a basis for a speculative suggestion, which would at least to some extent mitigate the apparent deficiency of Hilbert’s criterion of simplicity: Hilbert may have intended, by developing such a theory of calculation as projected in this entry, to identify *all* fundamental cognitive operations the human mind is capable of carrying out; the precise analysis of the deductive structure of mathematics Hilbert championed in the guise of what he called “the axiomatic method” may have also been intended to serve this overarching, philosophical purpose.

One may call this aim: “the epistemological analysis of mathematics”^52^; alternatively, one could label this goal the “logical analysis of mathematics”, where “logic” is understood not in the sense of “mathematical logic”, but rather in the more comprehensive sense current throughout the $19\text {th}$ century, i.e. as meaning “the codification of the activity of the faculty Kant called ‘Verstand’”.

In this sense, Hilbert appears to have been and remained throughout his career, a “logicist”.^53^

What distinguishes this position I am imputing to Hilbert from the kind of logicism traditionally associated with Russell or Frege, as I see it, is that Hilbert did not take a certain conception of logic – involving, amongst other things, a primitive capacity for unrestricted class-comprehension – as a starting point for reconstructing mathematics; rather, Hilbert took mathematics, as he found it, as his starting point and believed that the careful analysis of the deductive structure of mathematics would eventually reveal what the fundamental cognitive capacities of the human mind must be.^54^

Some support for this speculative hypothesis comes from an entry found on the loose sheets inlaid at the end of the third notebook, where Hilbert writes:

Our rules: substitution, abbreviation, append arguments (also to propositions) are complete and no extension is possible. Because our thinking is exhausted by these actions and is finite (no magic!)^55^

If Hilbert did indeed pursue some such overarching philosophical project, the criterion of simplicity he puts forward in the sketch of his $24\text {th}$ Problem could be satisfactory, *if* supplemented by such a theory of calculation as projected in the second notebook (see page 16): Since all proofs must, ultimately, be analysable in terms of the same (finite) number of fundamental cognitive operations, merely comparing the number of these operations deployed in a given proof (and, if some order of relative simplicity is introduced for the fundamental operations themselves, the number of specific operations used, rather than others) is a workable measure for the simplicity of the proof.

### Hilbert’s theory of problems

Whilst only some of the questions about mathematical problems raised in the passage I quoted on page 14 reappear in Hilbert’s sketch for his $24\text {th}$ Problem, Hilbert did also pursue some of the others further in subsequent notebook entries, but not only there: Hilbert took up, in particular, that entry’s apparent centrepiece – that with every problem is associated what Hilbert calls a “superordinate problem” (“Oberfrage”/“Oberproblem”^56^) which, according to Hilbert’s sketch in this entry, generally consists in asking whether the problem in question has a positive solution at all or is impossible to solve – again later. I will now survey these developments, which shine yet more light on Hilbert’s thinking about mathematical problems from yet another new direction.

In several entries Hilbert articulate the idea that mathematical problems arise by nature in some fixed order; e.g. on p. 50 ( in the entry immediately preceding the lengthy entry I quoted and before introducing the terminology I translated as “superordinate problem”), Hilbert claims:

Whenever a mathematical problem, I mean a question *A* has been posed, then one can deal first with the question whether this question *A* can be dealt with by means of a finite number of operations or not. Call this question *B*. From this in the same way question *C*, *D*,... The current state of the science determines where this chain of questions breaks off. [...]^57^

This idea of questions “stemming” from others in a fixed order subsequently gives rise in Hilbert’s mind to the idea – expressed in the following entry from the third notebook – of an immediately superordinate question, which, Hilbert appears to claim, *must* be solved beforehand^58^:

Amongst 100 thoughts, which are in principle equally good, only one may have lasting success. But which one it is you have initially no grasp on. You must think through a matter until you can say quite clearly why you are making no headway, i.e. what prior work, which can be carried out but is, for the time being, too difficult, must be dealt with beforehand in order to solve the problem posed, i.e. you must at least solve the next superordinate problem $B=Q^2$.^59^

Note that whilst Hilbert speaks of “superordinate problems” here, he seems to have in mind not specifically questions asking whether a given question can be solved by means of finitely many steps, but rather all kinds of problems whose solution might contribute to the solution of a given problem. By the time Hilbert began writing entries into the third notebook (likely around 1900), Hilbert thus appears to have expanded the idea of a « natural order »amongst problems to comprise not only questions asking, specifically, whether other questions can be solved at all, and how much calculatory work is required to that end, but also all kinds of (unspecified) “easier problems”:

Often, when a problem thoroughly resists your attempts of solving it, that is because you have not yet solved another, more proximate one: Were you to attempt to take off your socks and then take off your boots it will be impossible, but in reverse order it can quite easily be done.^60^

Although Hilbert never dedicated any publication to the matter after 1900, Hilbert did not keep his general reflections on problems – for which Hilbert coins the label “theory of problem-statements” (“Theorie von Problemstellungen”) in the third notebook^61^ – entirely to himself: A relatively extensive report of a presentation on the topic of “mathematical problems” given by Hilbert on December $21\text {st}$ 1910 is published in the *Jahresberichte der Deutschen Mathematiker Vereinigung*, which reports Hilbert laying out “some general considerations” about “superordinate problems” in the sense first outlined in the lengthy entry from the first notebook, i.e. the problem whether a given problem can be solved by means of finitely many operations.^62^

## Finitude and decidability

This preoccupation with finitude features, sometimes more and sometimes less prominently, in many of the contexts where Hilbert discusses mathematical problems in general terms, not least of all in the few places where Hilbert communicated some of his reflections about mathematical problems, long ongoing in private, to a wider public: In the $10\text {th}$ Problem of Hilbert’s list presented in 1900, Hilbert raises the challenge of “*exhibiting a procedure by way of which one can decide through a finite number of operations, whether an equation in whole rational numbers is solvable*”.^63^ Later, in “Axiomatic Thought” (1917) the “problem of *decidability* of a mathematical question by means of a finite number of operations”^64^ is raised, amongst several other examples metatheoretic questions (including “furthermore the question concerning a *criterion for the simplicity* of mathematical proofs”^65^). This preoccupation with the finitude of the number of steps makes its first appearance in the entry from the first notebook which I quoted on page 11, where Hilbert asserts:

Every [...] decision is possible by way of a finite number of operations (calculations).^66^

### Hilbert and Kronecker

In several entries scattered throughout all three notebooks, Hilbert associates this concern about finitude with the name of Leopold Kronecker. So, for example, Hilbert speaks of “the Kroneckerian requirement that everything must be accomplished by a finite number of processes”^67^ or of “the finitude of executable operations” in the following entry from the second notebook:

Concerning the finitude of executable operations in mathematics. Kronecker’s view that the proof of this finitude is what provides a secure grounding for a concept is not logical. One could rather say that this proof is necessary so that anything falls under the concept.^68^

Whether Kronecker actually made any such demands is doubtful. Kurt Hensel, in the introduction to his edition of Kronecker’s lectures, does report that “[Kronecker] was of the opinion that one could and must formulate all definitions in [what Kronecker called ‘general arithmetic’] in such a way that a finite number of trials would suffice to check the applicability to a given magnitude”.^69^ But, as Hensel emphasises only a few lines later on the same page:

Kronecker was a far cry removed from rejecting totally a definition or a proof that did not conform to these highest requirements, but he thought that in such cases something was still missing and regarded the supplementation in that direction as an important task.^70^

Since Hilbert met and spoke with Kronecker in person on at least one occasion,^71^ it is, of course, possible that Kronecker made some more definitive assertion in conversation, which Hilbert is accurately reporting in this (and similar) passages;^72^ on the other hand, it is not out of the question that Hilbert is unwittingly misremembering or intentionally (e.g. for rhetorical or dialectical effect) misrepresenting Kronecker’s position, which is, in fact, fully and accurately summarily by Hensel.

Personal animosity against Kronecker may have played a role in Hilbert’s contrarian attitude towards any methodological claim he associated with Kronecker: In the first notebook Hilbert records (on pp. 53f.) reports he has received “from different sources” that “Kronecker himself supposedly said” that “my investigations were only an extension of Kronecker’s ideas to invariant theory”. That Kronecker really did make such a claim seems plausible also in light of Hilbert’s (earlier) report of his encounter with Kronecker in Berlin in 1888, where Hilbert reports Kronecker saying that “[...] with his characteristic everything was done (work from the year 1878). Harnack’s work, too, as well as my future investigations [...]”^73^ These reports lead Hilbert to the – relative to his normally calm and factual tone – rather forceful assertion that the relevant “methods and results are my property (...) and I will let nobody take a jot of them, whoever he may be.”^74^ What little direct correspondence with Kronecker survives amongst Hilbert’s papers^75^ also shows that there was a further misunderstanding – or posturing – between them about sending each other offprints of their work, which Hilbert did but Kronecker did not to do—because, as Minkowski told Hilbert after a visit to Berlin,^76^ Kronecker thought (or claimed) that Hilbert had neglected to do so. (After Hilbert sent Kronecker a letter alerting him to this “misunderstanding”, as Hilbert calls it in a draft-letter[76], Kronecker sent to Hilbert (along with a letter dated 4/VII/1890) “4 parcels” of offprints, which Kronecker claims – whether truthfully or not is, of course, impossible to determine – had long been prepared but not posted due to “an extraordinarily incomprehensible mistake”^77^.)

Regardless of whatever ill will Hilbert harboured towards Kronecker personally as a result of these events, and of any (more or less well-founded) misgivings he had about Kronecker’s methodological standpoint in the early stages of his career (as document in the notebooks^78^), Hilbert seems to have eventually come around to Kronecker’s demands, as shown by a note, which reads:

Language alone without experimentation is incomprehensible. Therefore Kroneckerian decidability must also be present.^79^

Dating this note, and Hilbert’s embrace of “Kroneckerian decidability” along with it, with any certainty is impossible since the note is written on a loose sheet, not dated, nor firmly embedded in a context that gives any clear indications about its date of composition. However, the fact that these two sentences are flanked (on the sheet) by remarks reminiscent of Hilbert’s efforts, carried on continuously from 1905 until 1922, to find a satisfactory resolution to Richard’s Paradox, suggests that this note dates most probably from the mid-1910s,^80^ i.e. from a time (shortly) after Hilbert filled up the third notebook and started taking notes on loose sheets instead (see footnote 7).

That Hilbert arrived at this concession to Kronecker’s methodological viewpoint shortly after the third notebook was full is corroborated also by one of the last entries in the third notebook, where Hilbert labours to articulate a sense of “experimentation” that would be applicable to language, in line with the cryptic remark about “language without experimentation” above:

The points, lines and planes of my geometry will never be anything other than objects of thought and never have anything to do with real points, lines, planes. The situation is entirely different with my things “or”, “and”, “not” . That is a spice unique in all of science, which lends these investigations about thinking a peculiar allure, that here suddenly the “and”, “not”, “or” have the meaning of the real “or”, “not”, “and”, since we, as it were, experiment with these things on paper, whilst we never could experiment with points, lines, planes.^81^ This is the region where the line between, broadly speaking, sensual experience and purest thought intermix and one does as a logician the same thing as the geodesist does in geometry, the experimentor in electric theory, namely to experiment with the things “or”, “and”, “not” on paper.^82^

A similar remark can be found in the first volume of the *Grundlagen der Mathematik*, where the idea of “carrying out thought-experiments with visually present objects” is, in fact, used to characterise Hilbert’s “‘finitist’ standpoint”.^83^ The presence of such remarks in the *Grundlagen der Mathematik* show that the note about “Kroneckerian decidability”, whatever its date, does express the substance of Hilbert’s later, considered position. Indeed, the *Grundlagen der Mathematik* mention none other than Kronecker, with implicit approval, as “the first to emphasise the demands of the finite standpoint”^84^; (if I am right in dating Hilbert’s embrace of what he calls “Kroneckerian decidability” in the note I quoted to the mid 1910s, this would show that Hilbert began to stake out the “finitist standpoint” well before the beginning to publicise it in the 1920s; cf. footnote 83).

At any rate, Hilbert apparently came around at some point to accepting – perhaps in spite of himself, if he held on to his Kroneckerian grudges – the indispensibility of what he calls “Kroneckerian decidability”, at least in some form. Note, however, that a gap remains between the “demands” Hilbert attributes to Kronecker and the core tenets of the “finitist standpoint” as described finally in the *Grundlagen der Mathematik*: Hilbert attributes to Kronecker, like Hensel does, a concern mainly with the finitude *of the number* of operations involved in checking the applicability of a definition or calculating a result; finitism, as Hilbert and Bernays come to characterise it, is concerned, rather, with what one might call the “inherent finitude” of the fundamental operations themselves, which, as (Hilbert and) Bernays explain(s) “take place within the bounds of concrete inspection”^85^; i.e. with the (in some sense) « intuitive »or « effective nature »of these operations.

However, upon closer inspection, such a concern with the « nature »of the fundamental operations, not just with their number, does shine through some of the remarks I have highlighted. So, for example, in the notebook-entry I quoted in footnote 67: Hilbert objects there to the requirement that “everything must be accomplished by a finite number of processes” by raising the counterexample – anachronistically speaking (if I understand Hilbert’s compressed remark properly) – of a generally but not primitively recursive property: whether (for given $a, b\in \mathbb {N}$) there exists an *x* such that $ax^3+b$ is a prime number. In a sense, solving this problem requires only finitely many operations (one takes the least *x* such that $ax^3+b$ is prime and runs through all (finitely many) numbers $1<n<ax^3+b$ to verify that *n* does not divide $ax^3+b$ without remainder, or if there is no such *x* for given *a*, *b* let *x* be a default-value (e.g. 0) signalling a negative answer), but in another sense the property is not « finitely solvable »because simply considering the least number *x* such that $ax^3+b$ is prime is not an « effective »(in the sense of “primitive recursive”) operation.^86^

Hilbert thus seems concerned about “the finitude of operations” in at least two different senses: Hilbert worried, as did Kronecker, about the finitude of the *number of operations* in the third notebook (see footnote 67) after worrying – albeit still inarticulately – about the « inherent »finitude of the *operations themselves* already in the counterexample to Kronecker’s demand sketched in the second notebook (see footnote 68). Concern with the former sense of finitude – of allowing only *finitely many* operations – likely faded into the background with the advent of precisely defined artificial languages and deductive systems (from 1817/18 onwards), when Hilbert settled matter for himself once and for all: The codification of the formation rules of such languages enshrined the requirement that the number of steps in a calculation must always be finite in the formation rules for function-terms (which admit any finite number of nested operations, but no infinite number); the precise definition of a proof of a well-formed formula of such an artificial language did the same for the number of steps in a proof. This left the second sense of finitude – of allowing only « *inherently* finite »operations – to take center stage in the run-up to the *Grundlagen der Mathematik*.

Yet another, different or at least specifically distinct sense of “decidable” entered the picture in the early 1920s when Heinrich Behmann articulated what he called the “Entscheidungsproblem”.

### The decision problem

The problem Behmann formulated centred on the question whether it was possible, given a precisely defined deductive system, to describe a procedure for producing a proof of any not yet settled proposition or its negation *in a uniform fashion* that is the same for all open problems formulatable in the same artificial language as the deductive system is couched in^87^ The decision-problem as articulated by Behmann is thus more demanding than the requirement that proofs have finitely many steps, and more specific than the requirement that to be admissible in mathematics operations must (in some sense) be « effective »: Behmann is not concerned about the effectiveness of the mathematical operations invoked in individual steps of a proof, but rather about the effectiveness, in particular, of the « logical »operation involved *in exhibiting the proof* itself.^88^

Hilbert appears to be well aware of this difference when, in his 1921/22 lectures, as recorded by Kneser, he formulates Behmann’s decision-problem not as the problem of deciding *any given question* by a finitely long procedure or by means of « inherently finite »operations, but as the question whether “one can by way of a finite procedure decide whether a given formula is correct”.^89^ Though Behmann is not mentioned in the discussion of the decision-problem in the *official* notes to Hilbert’s 1922/23 lectures (produced by Bernays; Hilbert first lays out the decision-problem that year, thus after the publication of Behmann’s article in 1922), his name appears in *Kneser’s* student notes for that lecture-course and thus was presumably mentioned by Hilbert in the lectures.^90^

The origin of Behmann’s *Entscheidungsproblem* is, to an extent, obscure, but it seems to me some scholars have been overeager to ascribe to Hilbert a central role in raising it or even attribute Behmann’s *Entscheidungsproblem* to Hilbert outright,^91^ citing as evidence either Hilbert’s “No ignorabimus!”-slogan^92^, Hilbert’s $10\text {th}$ Problem or Hilbert’s preoccupation with decidability by finitely many operations in 1917 (see footnote 64). I have emphasised, to the contrary, that one can and should distinguish different senses of “decidable” Hilbert was, at different times, considering:

(I) The sense relevant to Hilbert’s conviction that all mathematical problems can, if they are clearly formulated, be solved either positively (by means of a proof) or negatively (by means of an impossibility-proof; cf. footnote 16). Whilst this conviction furnishes a sense in which Hilbert may be said to be concerned with “decidability”, in his thinking about the, as it may most aptly be called: “in principle decidability” of mathematical problems, Hilbert is concerned neither with the finite number, nor the finitary « nature »of the operations involved.

As I have argued in the first half of this article, Hilbert conviction of the principle decidability of all of mathematics – by means of “pure thinking” – is the result of a serious engagement with Kantian philosophy of mathematics; as such, it is originally totally separate from any concern with the finitude of operations, which Hilbert starts to think about when reckoning with Kronecker’s methodological standpoint and which leads in due course to:

(II) The sense relevant to the requirement that calculations should involve only finitely many operations (and proofs only finitely many steps). Applying the word “decidable” in this case is, upon reflection, misleading, given what “decidable” has since then come to mean; it seems to me that it would be better to speak instead of “(explicit or finite) definability” (in an artificial language), when computation is at issue,^93^ and to speak of the “finitude of proofs” when proofs are at issue. Because the recursive definitions (of well-formed formulae in an artificial language and proofs of such formulae within a deductive system in such a language) are what renders statements of what is sometimes called the “syntax-theory” of the language/deductive system decidable, one might speak in this sense of “syntactic decidability”.

As I suggested on p. 27, thinking in more depth about Kronecker’s strictures and their effect in practice, may have led Hilbert to think about decidability in yet another sense, which later becomes crucial in the characterisation of the finitist standpoint:

(III) The sense relevant to the claim that all (fundamental) operations in mathematics should be “inherently finite”, i.e. « effective », i.e. (primitive-(?))recursive operations.^94^ One may speak of this sense consequently as “effective decidability”. Hilbert, as I pointed out, also associates this claim with Kronecker when he labels it “Kroneckerian Decidability” in the note I quoted in footnote 79; this label (even if perhaps meant as something of a conciliatory honorific) is somewhat of a misnomer, however, since Kronecker himself seems to have been concerned mainly with (II) (and, indeed, Hilbert himself mentions Kronecker in his notebooks mainly in connection with (II)).

In Hilbert’s considered standpoint, as expounded (by Bernays) in the *Grundlagen der Mathematik*, *both* (II) and (III) are at play: The former codified in the formation rules for artificial languages and the precise definition of a deduction, the latter in the philosophical adumberation of the “finite standpoint”. Both taken separately go beyond (I), and, even in combination, these two sense of (a concern with) finitude and decidability remain separate from

(IV) the (broadly speaking: meta-logical) sense relevant to Behmann’s decision problem, i.e. whether the process of exhibiting a proof in a precisely defined deductive system couched in a strictly regimented artificial language is itself « effective ». Due to the centrality of the Entscheidungsproblem in Behmann’s sense in metamathematical research during the 1920s and 1930s, the word “decidable” has come to be used primarily in this sense; this is why I proposed to use it only with clarificatory qualifications (or not at all) for the other sense of (finite) decidability with which Hilbert was also at times concerned.

As I emphasised already, the last of these senses – “Behmannian Decidability”, one might call it – is decidedly different from (III) and certainly from (II) and I see no indication that Hilbert grasped it even vaguely before Behmann brought it clearly into focus.^95^ Formulating the Entscheidungsproblem is a stroke of originality for which, I think, one cannot but credit Behmann. About when and how, exactly, Behmann’s Entscheidungsproblem took shape in his mind little can, as far as I can see, be said or known for certain: Some kernels of it can arguably be gleamed from Behmann’s dissertation,^96^ but beyond that the process of invention remains a mystery. Behmann does not, in any case, credit Hilbert, or Hilbert’s work for decisive inspiration (although he credits Hilbert with presenting in lectures a decision procedure for sentential logic by means of normal forms^97^)^98^; instead, Behmann highlights Ernst Schröder’s work as a key precursor to his own.^99^

Not only should, I believe, these different sense of decidability be distinguished, one should also not assume that Hilbert saw connections between them – at least not (as, for instance, in the remark quoted in footnote 92) without serious argument – because, as I have been at pains to point out: Hilbert’s preoccupations with decidability in these different senses have distinct historical roots.

## Concluding remarks

Whether or not my interpretations are correct in spirit or in detail, all the evidence I have surveyed, in any case, shows how thinking in general (and philosophical) terms about mathematical proofs, problems and about the question(s) whether one can in every (particular) case *decide* whether a properly formulated claim holds or not, was close to Hilbert’s heart already long before his address to the World Congress in 1900 and remained there throughout most of his career, in one guise or another: In the beginning, Hilbert was led, I have argued, by his reading of Kant (documented in the first volume of Hilbert’s *Wissenschaftliches Tagebuch*) to reflect on and accept the, so to speak: “in principle”-solvability of any mathematical problem (so long as it is properly formulated) without taking any specific stand on *what means* were admissible or (in)sufficient to obtain that resolution (in a given case). In this respect, Hilbert’s views developed substantially in the course of time: In spite of his thoroughly ambivalent feelings about Kronecker personally, as well as about Kronecker’s methodology (as Hilbert construed it, recall the reservations in the scholarship I cited in footnote 70), Hilbert eventually came to accept also that the solution of any given problem must be obtained by means only of operations which are, in some sense, « inherently finite ».

Whether every mathematical problem can, *in principle*, be settled – as Hilbert was convinced it could since the beginning of his professional career –, even that it can be settled by way of finitely many steps, is, on the face of it, altogether different from the question whether this can be accomplished *in an effective manner*.^100^ Indeed, Hilbert appears, before eventually warming up to Kronecker’s methodological standpoint, to have been thinking mainly about what I called “‘in principle’-solvability”. (I think this describes, in particular, Hilbert’s position in 1900 when he delivered his address in Paris, and maybe even still Hilbert’s position when he delivered and his lecture “Axiomatic Thought”, which, I have argued (in footnote 65), is mostly retrospective.)

But, as the following passage from the third volume of Hilbert’s notebooks shows, Hilbert eventually came to think of these various senses of “decidable” as somehow related (and does so again with Kant’s work apparently on his mind, since Kant’s name is mentioned on the same page):

The properties of thought 1.) [that] only that is rigorous and correct which is secured as it were by experimentation like $2+1=1+2$. 2.) That every mathematical proposition is provable, are certainly more deeply related and flow from a common source.^101^

Whilst Hilbert does not venture any suggestion as to what this supposed common source might be,^102^ Kant’s philosophy of mathematics, as Hilbert came to understand and accept it, can furnish an answer: If mathematics, according to Hilbert (following Kant), deals in pure thought, then *the effectiveness*, which Hilbert came eventually (following Kronecker, perhaps at first only unwillingly) to accept *as a requirement all fundamental cognitive operations must conform to* is the shared source of the (effective) solvability of all problems and the standard of truth as (effective) calculability.

Hilbert’s preoccupation with mathematical problems neither began, nor ended when he chose – for whatever reason^103^ – not to present the issue as a $24\text {th}$ Problem in his Paris address; indeed, the wealth of entries in his unpublished notebooks dealing with questions and issues related – one way or another – to the overarching ambition of erecting a mathematical theory of mathematics itself, its problems as well as its proofs, reveal a tangled web of ideas and projects, most of which – like Hilbert’s “Theory of Problem-Statements” or his “Theory of Calculation” – smack of philosophy and appear not to have progressed beyond more or less daring reflections on certain suggestive observations that struck Hilbert at one time or another; the connections between these various strands of thought and their most famous points of distillation – such as Hilbert’s staunch opposition to irremediable ignorance, the philosophical goals served by his axiomatic method and the emergence of what has come to be known as the “Decision Problem” in Heinrich Behmann’s work –, remain difficult to discern, although I hope to have made progress in untangling them.^104^

## Data Availability

No quantitative data is used. Archival sources are cited with permissen from the relevant archive. In case the archives make available the manuscripts I refer to freely and publicly online, a link is provided in a footnote.

## Footnotes

1 So, for example, Cormac McCarthy’s last *grand roman gaché*, *Stella Maris*, invokes Hilbert’s problems on p. 135 (of the edition published by Picador 2022) as one of the many unfortunately superficial incisions into the flesh of mathematics meant to cut its protagonist out as a full-blooded mathematician: “I went through Hilbert’s problems.”, Alice Western is made to say, “Not to solve them but to try to find out what if anything they had in common.”

2 Hilbert (1935, p. 298). Rowe (2018, p. 189) points out that Hilbert may have been inspired to give a lecture of such ambition by the example of Boltzman, who delivered survey lecture of the development of physics a year earlier.

3 Grattan-Guinness (2000) presents a concise survey of the event and its historical context.

4 Hilbert’s papers are all listed at https://www.sub.uni-goettingen.de/fileadmin/media/texte/spezialsammlungen/Nachl%C3%A4sse/Hilbert.pdf (checked 3/IX/2024).

5 Hilbert noted on the inside of the cover of the first notebook that he purchased it “in Leipzig im Winter 1885/86” (this note was likely added only significantly later, since Hilbert can apparently recall the time of purchase only approximately; the note is also, unlike the first few entries in the notebook, not written in the old style of handwriting known as “Kurrent-Schrift”). Whether Hilbert began actively using it as a notebook immediately or only after some time is hard to say for certain since none of the entries are dated. However, that Hilbert spent the winter 1885/86 attending Klein’s seminar and writing a “Protokoll” for it (as reported by: Blumenthal 1935, p. 392) in concert with the mention (on p. 2 of the first notebook) of a “topic set by Klein” suggests that at least the first few entries were indeed written that winter, i.e. in the first few months of 1886. In any case, that the second notebook is dated (also on the inside of the cover) “12. November 1892” indicates that by late 1892 Hilbert had filled the first notebook.

6 One of the last entries in the third notebook (on p. 97) mentions a letter from Einstein to Sommerfeld “vom 29.10.1912”, from which Hilbert (to whom Sommerfeld must have given the letter) excerpts a few lines. The letter in question has since been published in the edition of Einstein’s correspondence (see: Einstein 1994, pp. 505 sq.), where the editors note that Sommerfeld “appended a letter to David Hilbert of 1 November 1912”. This indicates, that Hilbert saw Einstein’s letter soon after Sommerfeld received it. On the (probable) assumption that Hilbert excerpted it into his notebook on that occasion, this reference indicates that the third notebook was nearly filled in late 1912 and so probably finally full in early 1913 at the latest. In any case, as far as I see, no references to anything clearly later than 1912 are to be found in the notebooks; the loose sheets found in back of the third notebook, designated by the SUB as “Cod. Ms. D. Hilbert 600/3:Beilage”, are no exception, though they are (at least in part) clearly much later, as a mention of Hitler on fol. $2^v$ demonstrates, since there is no reason to think they were originally part of the notebook, or that it was Hilbert who placed them there, or, if he did, that he did so for any substantive reason.

7 There are only three such notebooks and no reason to think further volumes existed but were subsequently lost. In the first notebook a summary prospectus can be found (written by Hilbert on a loose sheet, folded up and placed just after the cover (the paper has since fallen apart into four pieces)) where Hilbert compiled references to what he thought, retrospectively, were the most important entries throughout all three notebooks (referred to by Roman numerals); if Hilbert had kept further notebooks, one would expect them to be at least mentioned in this overview. After 1913 (or whenever the third notebook was full), Hilbert appears to have shifted his note-taking away from dedicated notebooks to loose sheets – often page-proofs for the *Mathematische Annalen* –, which he either kept as such (see, e.g. the convolutes Cod. Ms. D. Hilbert 679–681; Cod. Ms. D. Hilbert 600/3:Beilage also consists of such later “Notizzettel”) or placed between the pages of thematically related lecture-notes; Hilbert also jotted down notes in the margin of lecture-notes, likely when revisiting them to teach once again the same or a related course. That many of the loose Notizzettel must date from a later period in Hilbert’s career is clear on account of the topics they discuss (e.g. the $\varepsilon$-calculus Hilbert developed in the 1920s) or names (like “Brouwer”) they mention.

8 The notebooks are paginated by Hilbert (though only on odd-numbered pages). This entry is found on pp. 25f. Until I systematically read through relevant scholarship in preparing this article, I was unaware that Rüdiger Thiele had already publicised this additional Problem – I had found it, like I assume Thiele did, whilst reading though Hilbert’s notebooks – in his Kenneth O. May Lecture in 2000 (later published as Thiele 2005). Though I think Thiele is exaggerating when he calls this “the most interesting problem of Hilbert’s collection” (Thiele 2003, p. 2), it still surprised me that Thiele’s publication of this fragment (along with a generous selection of other notebook-entries quoted throughout Thiele 2005) did, so far as I am aware, not ignite any more interest in Hilbert’s notebooks, which contain many more interesting entries besides this one; perhaps part of the reason is that Thiele’s own discussion did not convey a clear enough sense of the notebooks as cohesive documents of Hilbert’s intellectual development.

9 I am omitting here a second example Hilbert outlines – so-called “syzigies”, a concept pertinent to Hilbert’s early work in invariant theory –, which is discussed extensively by Thiele (2005, pp. 285 sq.).

10 The German text has been published, usually accompanied by an English translation, several times since Thiele initially publicised it. I want to highlight especially the most recent publication by Hipolito and Kahle (2019, pp. 1 sq.) because images of the manuscript are here reproduced alongside a transcription and translation [apart from the images and the literature-survey on pp. 6f., this article is substantially a reiteration of Kahle and Oitavem (2018)]. Digitisations of all three notebooks are freely available from the Göttinger Digitalisierungszentrum: https://gdz.sub.uni-goettingen.de/volumes/id/DE-611-HS-3180969 (checked 3/IX/2024).

11 Thiele (2003, p. 5) points out that in light of entries found a few pages earlier, which discuss Blumenthal’s habilitation, this entry was likely written in 1901 (at the earliest; even where some entries can be dated on the basis of circumstantial evidence, it is hard to derive from that precise dates for subsequent entries, especially in the third notebook, which Hilbert appears to have used for more than 10 years (see footnote 6), so significant amounts of time may, on occasion, have passed between one entry and the next). The earliest hint towards such a mathematical proof-theory in Hilbert’s *published* writings is Hilbert’s speaking of “considering a proof as itself a mathematical object” in Hilbert (1904, p. 261), well after 1900 (or 1901-or-so).

12 That this issue was the core of the $24\text {th}$ Problem as Hilbert saw it is suggested by the fact that Hilbert summarises this entry as part of the prospectus of « highlights »to be found in the three notebooks (see footnote 7) as “Gibt es einen einfachsten Beweis? III S. 25”. (Thiele and Wos 2002, p. 73) assert that Hilbert “made and inserted” the prospectus “at the end of his life”, but cite no evidence, as, indeed, so far as I can see, *there is no evidence* to pinpoint the time of composition of the prospectus found in front of the first notebook. It certainly *could* have been compiled in the 1930s, as Thiele conjectures, but it might just as well date from the 1910s, when the third notebook was full, or from any time in-between; Hilbert may even have started it before filling the third notebook and added to it over time. Thiele makes the same assertion also in Thiele (2003, p. 7); Hipolito and Kahle (2019, p. 3) cite Thiele’s claim as fact without questioning its evidential basis and take it to attest that Hilbert remained concerned about simplicity throughout his life. There is, in fact, little firm evidence to suggest that Hilbert thought much and seriously about simplicity in the 1920s or thereafter (or, indeed, earlier).

13 See the editor’s introduction to the relevant lecture notes in Hilbert (2013, pp. 418–430), where it is emphasised, in particular (on p. 423) that Hilbert’s lecture-course in 1922/23 “represents quite a new direction for proof-theoretic investigations”.

14 Hilbert (1935, p. 297).

15 A testament to how lasting an impression this particular result made on Hilbert – one may speculate that part of the reason is that establishing the transcendence of $\pi$ was the main claim to fame of Hilbert’s own doctoral supervisor, Ferdinand Lindemann, who may have impressed on Hilbert the importance of his discovery; Hilbert’s continued interest in it, and in alternative proofs superseding Lindemann’s own, could thus serve Hilbert also as a way of reckoning with his own past – is the fact that he lectured on the “Problem of the Quadrature of the Circle” throughout his career (see the list of Hilbert’s lectures compiled in: Hilbert 2013, pp. 993–1009), first in 1894/95 and for the last time in 1928/29. Over time, Hilbert turned these lectures into a kind of catch-all survey-course about the organisation and methodology of mathematics as a whole – in 1924/25 Hilbert even adopted as a subtitle for this lecture-course what had been the title of Felix Klein’s survey-lecture-course, “Elementarmathematik nach höheren Methoden” (see p. 1004) –, as his introductory remarks for his lecture-course in 1914/15 attest, where Hilbert declares his intention to “let matters of principle emerge by relying on special [...] classical problems [like] [the] quadrature of the circle” (Hilbert’s preparations for this course, including his opening remarks, are preserved in a notebook held under the signature “Cod. Ms. D. Hilbert 559” by the SUB Göttingen, see fol. $29^v$; an anonymous set of lecture notes taken by a student exists at the Library of the Institute of Advanced Study at Princeton (a scan of which is now freely available through the Library of the IAS’ website: https://hdl.handle.net/20.500.12111/7950 (checked 30/12/2022), p. 3.). This course is worth highlighting also because it shows how Hilbert’s choice of titles for his lecture-courses obscures the prevalence of the quadrature-problem in his teaching: The 1914/15 lecture-course does not mention the quadrature of the circle in its title (it is entitled “Elements and Questions concerning the Principles of Mathematics”), yet more than half of it (pp. 2–122 out of 218 pages of the anonymous Princeton notes) is devoted to discussing the quadrature of the circle; Hilbert’s lectures in the summers of 1908 and 1910, which also bear titles mentioning “principles”, were likewise devoted mainly to (demonstrating the impossibility of) squaring the circle.

16 Hilbert first discusses impossibility-proofs (“Unmöglichkeitsbeweise”) systematically, as far as I am aware, in a lecture-course delivered in the summer-semester of 1913, recorded in part in Cod. Ms. D. Hilbert 559, which contains a whole section recording such a discussion of “Unmöglichkeitsbeweise” on fols. $11^v$–$16^v$. In an entry on fol. $3^r$ of the sheets found in back of the third notebook (i.e. Cod. Ms. D. Hilbert 600/3:Beilage, likely written much later and coincidentally placed in the third notebook, see footnote 6) Hilbert credits Kant with being the first to make explicit impossibility claims in rejecting proofs for the existence of god.

17 Thiele (2003, p. 10) states that Hilbert “had preserved from his university days” this conviction, but since Thiele cites no sources, it remains unclear what aspects or passage in Hilbert’s (unpublished) writings or correspondence Thiele has in mind as a basis for this claim. The claim itself is, in my view, substantially correct, in any case, and my aim here is to bring to light its roots in Hilbert’s intellectual development.

18 “Vorausgesetzt, dass es keine praktischen Schwierigkeiten gäbe, ist der menschliche Verstand vermögend, alle Fragen, die er sich selbst stellt, zu lösen? Muss es möglich sein zu entscheiden über die Quadratur des Kreises, gibt es eine wohl definierte, auf die sinnlichen Dinge bezügliche Frage, welche eine Antwort haben muss und die gleichwohl sich nicht beantworten lässt. Kann man einen allgemeinen zwingenden Beweis führen, dass jede Erkenntniss möglich sein muss?” (Wissenschaftliches Tagebuch I, p. 32)

19 The phrase “Theorie der Logik-Arithmetik” is presumably a reference to Hilbert’s programmatic remark from 1904 that a “simultaneous development of the laws of logic and of arithmetic” is needed to obtain a satisfactory account of the foundations of mathematics (see: Hilbert 1904, pp. 250); (or, if this entry predates, as it very well may, 1904, it presages that programmatic remark Hilbert made in his address to the next International Congress of Mathematics after Paris 1900 held in Heidelberg in 1904).

20 The entry reads: “Dass es kein Ignorabimus in der Mathematik giebt, ist wahrscheinlich durch meine Theorie der Logik-Arithmetik beweisbar.” (Wissenschaftliches Tagebuch III, p. 104).

21 Later in the third notebook, one finds Hilbert using the phrase “proof of all proofs” in this sense: “Beweis aller Beweise: dass man den Beweis immer muss finden können!” (Wissenschaftliches Tagebuch III, p. 96), Hilbert does presumably not mean to (absurdly) assert that every proposition can be proved, but rather that one can find either a positive proof *or an impossibility-proof*; the point is, rather, that every question can be settled *rigorously*, whether positively or negatively. The phrase “proof of proofs” can also be found on sheet 22 in the convolute Cod. Ms. D. Hilbert 601, which, because it contain a lot of the same formulations as one finds on pp. 95f. of the third notebook, may date from roughly the same time. Thiele and Wos (2002, p. 74) assert that this convictions “rests on [the] philosophy of finitude” presented mainly in Hilbert’s “On the Infinite” (in 1925 (!)), but this seems to me an unfounded projection of ideas Hilbert promulgated (much) later into (far) earlier writings.

22 On p. 12 of the first notebook Hilbert takes issue with a position he explicitly attributes to Weierstrass, namely that “the order of a function in (homogeneous) variables is too insignificant to base theories on it”; Hilbert reports Weierstrass taking the position that “the degree, i.e. the order of a curve [is] something too insignificant to base a theory of reality-questions on it” on the page numbered (by an archivalist) “$1/6^v$” of the small diary Hilbert kept during his extensive travels in 1888 (i.e. Cod. Ms. D. Hilbert 741:1, which has recently been made freely available by the GDZ: http://resolver.sub.uni-goettingen.de/purl?DE-611-HS-3149147 (checked 28/II/2025)). This parallel suggests that entries after p. 12 were written after Hilbert’s encounter with Weierstrass in 1888, and since the first notebook was apparently full in 1892, when the second one begins, 1890 is a plausible conjecture, give or take a year, for an entry on p. 96. The label “reality-questions” (“Realitätsfragen”) appears several times in Hilbert’s scientific diary. In an entry on p. 48 of the first volume Hilbert even mentions a (by that point) “old ‘manuscript’” about reality-questions (which fits well with the fact that Hilbert would have talked about it with Weierstrass in 1888). I have not succeeded in identifying such a manuscript amongst Hilbert’s papers (it is likely lost), so I find myself reduced to reporting Hilbert’s bibliographic references (from an entry on p. 28 of the first notebook, and a list of references associated with the topic in Cod. Ms. D. Hilbert 741:14, where Hilbert mentions: Klein (both), Kronecker (diary), [Walther von] Dyck (diary), Friedrich Meyer (diary), Maxwell (741:14), Sylvester (741:14), Möbius (741:14) as a (slim) basis for guesswork as to what Hilbert may have meant by that label; judging by the references Hilbert associates with the topic, the manuscript was, in spite of its suggestive title, likely not philosophical in content or ambition, but belonged, rather, to Hilbert’s invariant-theoretic work of the 1880s (the references to Meyer and Sylvester, in particular, strongly suggest the investigations in question pertain to invariant theory, since that is the focus of these authors’ work).

23 The entry reads: “Begriff der ganzen Zahl beruht nothwendig auf Zeit und Raum. Begriff der Stetigkeit einfacher als als [sic, or “also als”?] der Begriff der ganzen Zahl Kant hat Recht.” (Wissenschaftliches Tagebuch I, p. 28). Hilbert does not specify what part of Kant’s writings he is thinking about, but a likely candidate is the discussion in Kant (1998, A169 sqq./B211 sq.), where Kant maintains that all appearances are continua, whilst “all number presupposes a unit”, which must be given as an appearance before it can be used as a unit to measure, e.g. the number of discrete objects (Kant’s example are coins); this suggests a view like the one Hilbert sketches, that continuity is conceptually prior to number, because one cannot conceive of a unit, which is the basis of the concept of number, without having already conceived of a continuum. (I don’t mean to endorse Kant’s argument, nor Hilbert’s interpretation of it, merely to point out that this is likely to be the passage Hilbert *is* reflecting on in the entry on p. 28.) A kindred view (that continuity is conceptually prior to what Kant calls “forms of intuition”) was articulated (with explicit but ambivalent reference to Kant) by Cantor in (1932, p. 192).

24 “In allen anderen Wissenschaften Material, von dem man nicht weiss, wo es herkommt, von dem man nur weiss, dass es da ist. Hier reines Denken, reine Philosophie. Kant hat das als erste[r] gefühlt, aber nicht bewiesen, er hat angefangen, aber nicht zu Ende geführt.” (Wissenschaftliches Tagebuch I, p. 28)

25 That Hilbert studied the section of Kant’s *Critique of Pure Reason* entitled “Transcendental Dialectics” closely is plausible not just because Hilbert took from there the epigraph for his *Foundations of Geometry* (1899), but also because of the remark found in the Beilage to the third notebook (see footnote 16), where Hilbert alludes to Kant’s discussion of proofs of the existence of god (see footnote 16), which also forms part of the “Transcendental Dialectics”. Further evidence that Hilbert studied, in particular, the section on antinomies is provided by the entry from the third notebook (p. 93), where Hilbert claims that “[t]he Kantian antinomies appear as simple errors in calculation within the logical calculus”. It is worth noting that on the page before this remark one finds Hilbert substantively reiterating a Kantian view of mathematics on p. 94 (entries are written in reverse, contrary to the pagination, on pp. 137–92) of the third notebook: “Math. ist Denken, reines Denken, nichts weiter d.h. bestimmtes, richtiges Denken, Denken schlechtweg.” This re-affirmation, in combination with the remark about antinomies as calculation mistakes, suggests that Hilbert was at this point re-reading, or at least recalling (parts of) Kant’s *Critique of Pure Reason*. In this article, in any case, I deal only with Hilbert’s uptake of Kant’s views of mathematics as a pure science of reason. In another forthcoming article, entitled “Philosophy of Mathematics before 1900: An Early Manuscript by David Hilbert” I discuss archival evidence of Hilbert’s engagement with Kant’s views of arithmetical propositions as “synthetic a priori judgements”—which Hilbert reaffirms, at least in the early manuscript discussed there (Cod. Ms. D. Hilbert 606), whilst denying the synthetic a priori status of the propositions of geometry (notably with explicit reference to Riemann’s work). I am currently preparing a final article on Hilbert’s engagement with Kantian philosophy, where I discuss the epigraph of the *Foundations of Geometry* and Hilbert’s philosophy of geometry.

26 “Die Mathematik gibt das glänzendste Beispiel, einer sich, ohne Beihülfe der Erfahrung, von selbst glücklich erweiternden reinen Vernunft.” (A712/B741). Indeed, it seems to me not implausible to interpret Hilbert’s formulation “[h]ere is [...] pure philosophy” (on p. 28 of the first notebook, quoted above) as a somewhat confused echo of Kant’s formulation of a “[science of] pure reason” (taking philosophy to be the paradigmatic exercise of reason).

27 Kant recognises two further pure sciences but assigns neither of them to the theoretical domain (see: Kant 1998, A480/B508), namely: what Kant there calls “pure ethics”, located in the practical domain, and what Kant calls “transcendental philosophy”, presumably conceived as some sort of overarching science of reason.

28 When labelling mathematics a “pure science of reason”, Kant moreover goes on to illustrate this status of mathematics by alluding to the same example of a mathematical question Hilbert invokes also: the squaring of the circle, although Kant cites Lambert’s proof of the impossibility of squaring the circle.

29 Kant (1998, A476/B504.)

30 Hilbert (1935, p. 298)

31 Rowe (2018, pp. 191 sq.) also briefly notes affinities between Hilbert’s thinking about mathematical problems and Kant, but does not discuss any of the evidence of Hilbert’s early engagement with Kant, whilst for the connection Rowe mainly highlights (concerning the status of the parallel postulate) there is no evidence that Hilbert saw any connection of his thinking to Kant’s on this particular point; on the other hand, Rowe sees no connection between Kant and Hilbert’s conviction that all problems are solvable, maintaining instead that “Hilbert took this simply as an article of faith”.

32 “Die Mathematik ist die einzige Wissenschaft, bei welcher wir nicht ignorabimus sondern im Gegenteil in weitesten Umfang im Bezug auf alle und noch so schwierige Probleme sagen müssen: *noszemus* [sic].” (Wissenschaftliches Tagebuch I, p. 72) (Thiele 2003, p. 8) refers to further, later expressions of this sentiment in Hilbert’s third notebook, but, for some reason, does not mention its first occurrence in the first notebook.

33 Wissenschaftliches Tagebuch I, p. 28.

34 The handwriting of this addition resembles Hilbert’s handwriting as one finds it in the third notebook much more closely, with respect to the size and shape of the letter, than it resembles the handwriting in Hilbert’s first notebook.

35 Reid (1996, p. 13) maintains that “to Hilbert and Minkowski such a concession [i.e. du Bois-Reymond’s ‘ignorabimus’] was thoroughly abhorrent” already during their student days, but since Reid’s only source for her assertion is a quotation from Hilbert’s 1900 address, this may just be an unfounded projection of the future into the past. I am not aware of any evidence that would decisively support Reid’s claim; as far as I can see, neither du Bois-Reymond – neither Emil, nor Paul – is ever mentioned in Hilbert’s notebooks. But I concede that, because there is scant evidence as to what Hilbert knew or read and when, my contrary claim that Hilbert learned (or seriously thought) about du Bois-Reymond’s dictum only after already becoming convinced that every mathematical problem, if properly formulated, could be resolved, is likewise only a hypothesis. On balance, I think my hypothesis fits somewhat better with the available evidence (so far as I can see), some of which I have highlighted, all of which is, however, insufficiently clear and detailed to definitively settle the matter one way or the other.

36 As documented, e.g. in: McCarty (2005, p. 68).

37 I take it Hilbert means “one in kind”, not “one in number”, that is to say, he believe that all mathematical problems take the form sketched earlier in the entry, of constructing a corresponding infinite sequences of 1s and 0s (subject, perhaps, to certain more specific conditions). The claim is still bizarrely strong and as far as I am aware, Hilbert never repeated it. Indeed, it is unclear on what grounds he is making it here. One might speculate that Hilbert is in this entry ruminating over Dedekind’s idea of a “fundamental faculty of our mind to relate things to things [...] without which no thought at all is possible” (Dedekind 1965, pp. III sq.). But Hilbert’s formulations aren’t strikingly reminiscent of Dedekind’s, and, as far as I see, there is no circumstantial evidence which suggests that Hilbert had read Dedekind’s foundational work with any seriousness at this stage in his career. There is an entry on p. 75 of the *third* notebook, where Hilbert appears to echo Dedekind’s formulation quite unmistakably, but even given the uncertainty involved in dating the entries of these notebooks, that entry would have been written more than 10 years later.

38 “Jedes mathem. Problem kann auf folgende Frage zurückgeführt werden: Gegeben ist ein Gesetz, vermöge dessen zu jeder vorgelegten nur aus Null und 1 bestehenden nichtabbrechenden Reihe $(\alpha ) ~ 0~0~1~1~0~0\dots$ eine bestimmte andere solche Reihe (durch Rechenoperationen: Grösste Ganze suchen, Teiler suchen, etc. nicht Würfeln...) $(\beta ) ~ 1~0~0~1~1~1\dots$ construirt werden kann. Man soll durch eine endliche Zahl von Operationen entscheiden ob in irgend einer Reihe ($\beta$) eine 0 vorkommt oder ob alle Reihen ($\beta$) nur Einsen bestehen [sic]? Ich behaupte: Jede solche Entscheidung ist durch eine endliche Zahl von Operationen (Rechenoperationen) möglich. d.h. Es giebt kein Gesetz, bei welchem die Entscheidung nicht durch eine endliche Zahl von Operationen möglich wäre. d.h. Jedes math. Problem ist lösbar. Alles dem menschlichen Verstande erreichbare (durch reines Denken ohne Materie) ist auch aufzulösen. Es giebt nur ein Problem. (z.B. Quadratur des Kreises. Hat $\pi =3,14\dots$ 10 aufeinander folgende 7en etc.) Von der Annahme der Möglichkeit geht man von vorneherein aus.” (Wissenschaftliches Tagebuch I, p. 37).

39 How committed Hilbert remained to this idea of mathematics as a “science of pure thought” also later is shown by an entry from the *third* notebook I quoted in footnote 25 from p. 94 of the third notebook.

40 There exists, in fact, an even more extensive document of Hilbert’s early engagement with Kant’s and Kantian views on mathematics put forward by various philosophers closer to Hilbert’s own time (e.g. Ueberweg and even Frege) in the manuscript held by the SUB under the signature “Cod. Ms. D. Hilbert 606”, which I believe to be a draft for a sort of « student-essay »Hilbert wrote in the summer of 1885 (see my article mentioned in footnote 25).

41 I take my discussion to show that Hilbert was – at least in this respect – more thoroughly and deeply a “Kantian” than has commonly been appreciated. Gray (2000, p. 14), for example, asserts that “it can be doubted if [Hilbert] was ever profoundly a Kantian” (unfortunately Gray does not elaborate on the evidential causes for such doubt). Gray’s book, incidentally, is a useful survey of the afterlife of Hilbert’s problems, of solutions and their failure, if one written for a somewhat chimerical audience, members of which are, on one hand, presumed to know (on p. 67) what “a commutative Abelian extension of *Q*” is more than a hundred pages before needing footnote 28 on p. 177 to explain: “A group *G* is commutative if and only if $gh=hg$ for every pair of elements *g* and *h* of *G*.”

42 As, for example, Rowe maintains (see footnote 31). Thiele (2005, p. 287) asks if Hilbert’s conviction that every problem was solvable was “part of the zeitgeist?”. In my view, Hilbert’s conviction owed less to what was generally « in the air »and much more specifically to Hilbert’s philosophical education, especially his engagement with the philosophy of Kant and Kant’s interpreters in Hilbert’s own time – as I hope to discuss in a future article, as described in footnote 25 –, in particular one of the professors of philosophy at Königsberg during Hilbert’s time as a student, Günther Thiele (1841–1910, professor of philosophy in Königsberg between 1882–1898, no relation to Rüdiger Thiele, as far as I am aware), whose *Grundriss der Logik und Metaphysik* (Thiele 1878) Hilbert quotes in Hilbert (2003, p. 120).

43 I emphasise that Hilbert in my view held the position I describe here *only* with respect to pure mathematics. I do not mean to claim, and do not believe, that Hilbert took such a position also with respect to physics and physical reality, as other idealist thinkers (in Hilbert’s own time, for example, Cassirer) did.

44 A similar contrast between Hilbert’s overall project and that of Intuitionism was drawn in a very prescient article by Abrusci (2000, p. 136).

45 This is not the only occasion, where Hilbert describes his colleagues as largely devoted to hopeless digging. In another, even bleaker simile recorded on p. 40 of the first notebook, Hilbert compares mathematicians to miners who carry on even after the shaft has caved in behind them: “Viele Mathematiker gleichen den Bergleuten, welche immer tiefer den Schachten graben, obwohl der Weg zu ihnen schon ver|schüttet ist, so dass keine freie Luft und keine neue Lebenskraft an die Arbeitsstelle geschafft werden kann: sie leisten für die Gesamtheit der Wissenschaft nichts und verkümmern schliesslich elend.” (Wissenschaftliches Tagebuch I, p. 40f.)

46 Hilbert’s original metaphor refers to the production of sculptures by means of moulds: Hilbert says the building of science should seem as if it required only a single cast (“wie aus einem Guss”), which is a popular metaphor in German but does not, as far as I am aware, have an equally prominent metaphor drawn from the same field of artistry corresponding to it in English, so I render it using a different but more prominent (?) sculpting-metaphor.

47 “Die Mathematik hat anderen Wissenschaften gegen|über den Vorzug, dass man bestimmt sagen kann, ob eine Frage erledigt ist oder nicht. – Schachspiel hat vor der Wissenschaft den bestimmten – freilich willkürlichen – aber ein für alle Mal gesetzten Zweck – das Matt-Setzen voraus, nach dessen Erreichung oder Verfehlung die Güte des Gedankens (der Methode) gemessen werden kann. In der Physik stellt die Natur die Aufgabe. Bei der Mathem. liegt die Gefahr des Fortrennens in der Willkür – der Thatsache entsprechend, dass der nämliche Verstand selbstständig aus sich heraus die Aufgabe stellt, sie löst und kritisch nach eignen Ermessen ohne äusseren Anhalt sie abschätzt und würdigt: Streben nach grossen leitenden Gesichtspunkten und die Vorschrift, die man sich selbst als Pflicht auferlegt, jedes einzelne Resultat in einer Beziehung zur Gesamtwissenschaft zu betrachten und zu vergleichen. Daraus ergiebt sich im Durcharbeiten des vorhandenen Stoffes besser als, wenn jeder seinen eigenen Mist weitergräbt, um den sich doch niemand sonst kümmert. Wer Referate macht, sieht, dass viele in dieser Weise die Wissenschaft treiben: Niemand zur Freude, sich selbst zur Qual. Nicht auf die Erledigung | bestimmter Fragen (diese nun als Leitmotiv, als vorschwe[be]ndes Ziel, als anreizendes Lockmittel) sondern darauf kommt es an, dass sich vor unserem geistigen Augen das stolze Gebäude der Wissenschaft harmonisch und einheitlich erhebt, wie aus einem Guss. Eine Frage *F*, für welche die nächste Oberfrage $F^2$ mit ja beantwortet wird, ist minder hoch zu schätzen – vielleicht Criterium für die Wichtigkeit einer Frage. Uebrigens ist es selbst wenn die nächste Oberfrage $F^2$ beantwortet ist, noch ein grosser Unterschied, ob ich bloss zeige dass etwas durch eine endliche Zahl von Processen möglich (wie bei meinem Beweis für die Endlichkeit der Invarianten ternärer Formen) oder ob ich wirklich eine obere Grenze, für die Zahl der Processe angebe, so dass es nur auf die Zeit und die Anwendung von Arbeit ankommt. Ferner ist auch noch die Frage, ob es Probleme in der Mathematik giebt, deren Erledigung nicht in einer vorgeschriebenen kleinen Zeit möglich ist? etc.” (Wissenschaftliches Tagebuch I, p. 51ff.)

48 Kahle and Oitavem (2018, p. 6) note as well that “it is rather questionable whether length would even be a good measure for simplicity” (this reservation and the surrounding discussion is reiterated, more or less word for word, in: Hipolito and Kahle 2019, p. 5). Issues with implementing Hilbert’s criterion are discussed (unfortunately in a style that exudes an often unbearable self-importance) by Thiele (2002, sec. 4), mainly with a view to giving an account of “simpler” proofs applicable in “proof-simplification” (p. 75), i.e. to estimate whether certain modifications of a given proof have made it simpler; it is not their ambition to compare alternative proofs that are not in any obvious sense modifications of one another, and none of the work they survey speaks to this issue. The comparison of proofs by different methods with regard to their simplicity is discussed by Arana (2017), who also survey some pertinent technical work.

49 Perhaps Hilbert’s somewhat puzzling formulation in the $24\text {th}$ Problem (see page 3) that any axiom of arithmetic “always corresponds to geometric propositions or logical inferences” indicates an inclination on Hilbert’s part (at the time) to regard geometric and logical reasoning as somehow fundamental for all of mathematics.

50 “Stelle auf eine Theorie des Rechnens d.h. ergründe worin die Vortheile bekann|ter arithmetischer Formeln und Sätze (Summation arithmetischer Reihen, Congruenzformeln, Stirlingsche Formel, vergl. Schlömilch Compendium Bd 2 S. 264.) zum Ausrechnen bestimmter Aufgaben beruhen und wie man diese Vortheile rechnerisch abschätzen kann. (Vielleicht indem man gewisse elementare Rechnungsoperationen (addiren, multipliciren, potenziren) als Einheiten einführt und die Zahl der anzuwendenden Einheiten auf verschiedenen Wegen, die man beim Rechnen einschlägt, bestimmt. Wie viel Rechnungseinheiten z.B. sind nöthig, um so und so vielte Stelle der Decimalbruch-Entwicklung von $\pi$ zu bestimmen? Wie berechnet man *n*! am schnellsten? Vielleicht die ersten Ziffern aus Stirlings Formel, und die letzten durch Congruenz-Rechnung.” (Wissenschaftliches Tagebuch II, p. [0]f., i.e. the very first (unpaginated) page preceding the page paginated “1”)

51 Passages like the one I quoted in footnote 38 suggest that Hilbert may have believed that, somehow, all (mathematical) thinking is ultimately a matter of computation. But even if Hilbert did hold such a view, he never articulated it explicitly, and there is also evidence suggesting that Hilbert opposed such assumptions: In other entries, such as the following, Hilbert appears to sharply rejects the idea that mathematics is essentially just calculation: “Was Gordan und Klein wollen, alles auf 7 Operationen zurückführen, das ist ja gerade die Absicht meiner axiomatischen Methode, so dass man jede richtige Thatsache in der Math. durch Erbsen beweisen, controlliren und jeden Fehler ebenso aufdecken kann. Nur der Anlass | die Anwendung meiner Th. ist eine andere, eine allgemeinere geistigere [sic]. Denjenigen, die Alles ausser Rechnen leugnen, die das Rechnen für etwas besonderes und das Ueberlegen, Begriffsbilden für etwas unerlaubtes halten, den Kroneckerschen Endlichkeitsfanatikern soll freilich durch meine Methode der Boden entzogen werden.” (Wissenschaftliches Tagebuch III, p. 102f., “eine allgemeinere geistigere” makes little sense as it stands, but it is unfortunately unclear how to fix the formulation, i.e. whether one should read instead “eine allgemeinere[,] geistigere” (i.e. supply a comma and construe both as coordinate adjectives: “a more general, more intellectual approach”) or, rather: “eine allgemeiner geistigere” (i.e. correct “allgemeiner” to an adverb modifying “geistigere”: “in general terms, a more intellectual approach”).). A reviewer has pointed out to me that Hilbert’s emphasis on thinking (“Überlegen, Begriffsbilden”) over calculating (“Rechnen”) signals, especially in a context where Kronecker is mentioned explicitly, Hilbert’s allegiance with the “Göttingen Tradition” of mathematics, and his opposition to its rival, the “Berlin Tradition” [for an overview, see Rowe (2000, p. 66); for the importance especially of “Begriffsbilden” in the Göttingen (=Riemannian) tradition, cf. Tappenden (2006, p. 112)].

52 The German word usually translated in English as “epistemology”, “Erkenntnistheorie”, was, indeed, widely used in this sense, i.e. as aiming at identifying and cataloguing the fundamental cognitive process of the human mind, in Hilbert’s own time. Hilbert was likely aware of this usage to some extent and, in an early lecture on the foundations of geometry, even ascribes mainly “erkenntnistheoretisch[e Bedeutung]” to the investigation of geometry by means of the axiomatic method (see: Hilbert 2003, p. 21).

53 Klev (2015, sec. 2) articulates a similar sense of “logicism” to rubricise Dedekind’s position.

54 Michael Hallett has long maintained that Hilbert saw his foundational work also as an exploration of “the actual workings of the mind” because Hilbert believed “that a (deductive) thought process can be resolved formally into finitely many atomic units” (Hallett 1994, p. 187); he has (in correspondence) brought to my attention a highly suggestive passage in Hilbert’s 1927 Hamburg lecture (recently reprinted in: Hilbert 2013, pp. 935 sq.), where Hilbert maintains that “the basic idea of my proof-theory is nothing other than to describe the activity of our mind [“Verstand”], to take notes about the rules according to which our thinking actually proceeds”. Even earlier, Kitcher (1976, p. 111) observed that both Kant and Hilbert “identify a process which we can recognise as occurring in our mental (mathematical) life and hypothesise that this process is the basis of our mathematical knowledge”. Besides Thiele’s work, Hallett’s article is the only one I am aware of to take into account (even only selectively) evidence from Hilbert’s notebooks (in endnotes 22, 40 and in the main text on p. 182). I want to make two remarks about Hallett’s reliance on the notebooks: First, Hallett states in endnote 40 that “Hilbert paginates [parts of the notebooks] in reverse”. The phenomenon Hallett thus describes is, I think, better described as follows: The notebooks are, in fact, paginated continuously (by Hilbert, always only on odd-numbered pages) from front to back, but in the second and third notebook Hilbert apparently maintained *two series of entries* in parallel: one (predominantly mathematical in content) starting in front, the other (predominantly philosophical in content) *starting in back* (in both notebooks this series of entries begins with a heading identifying it as containing “Allgemein Mathem. Philosophisches” (II, p. 107) and “Allgemein Mathematisch-Philosophische Bemerkungen.” (III, p. 137) respectively), so what is “in reverse” at the point Hallett is referencing is not the pagination but Hilbert’s way of adding entries relative to the notebooks’ continuous pagination. Second, whilst I am inclined to accept the dates Hallett proposes in endnote 40 (“between 1905 and 1910” for entries in the third volume) as approximately accurate, the date conjectured in endnote 22 (and presupposed on p. 182) of “a data [sic] around 1904–1905” seems to me too early for the entry in question, especially because (in light of the reverted order of entries) an entry on p. 101 would be (significantly) *later* than entries on p. 132–133 dated in endnote 40 between 1905 and 1910.

55 “Unsere Regeln: Einsetzungen, Abkürzungen, Argument-anhängen auch an Aussagen sind definitiv, vollständig und keiner Erweiterung fähig. Denn unser Denken geht in deren Handeln auf und ist finit (keine Hexerei!)” (Cod. Ms. D. Hilbert 600/3:Beilage, fol. $4^r$). Cf. the entry quoted in footnote 51; the prohibition of “magic” echoes the prohibition of determining the members of sequences by means of dice in the passage quoted in footnote 38.

56 Note that whilst the terminology Hilbert uses in these entries literally translates to “superordinate *question*” (“Ober*frage*”, not “Ober*problem*”), Hilbert himself (later) used “Oberproblem” as a synonym for “Oberfrage”, as the passage quoted in footnote 62 attests.

57 “Wenn ein math. Problem, ich will sagen eine Frage *A* gestellt ist, so kann man zuvor die Frage erledigen, ob diese Frage *A* durch eine endliche Zahl von Operationen erledigt werden kann oder nicht. Dies Frage sei *B* genannt. Hieraus ebenso Frage *C*, *D*,... Der jedemalige Stand der Wissenschaft entscheidet wo diese Kette von Fragen abbricht. z.B. Frage *A*: Giebt es $C_6$ mit 11 getrennten in Ovalen. kann ich bisher nicht beantworten. Dagegen sehr wohl die hieraus stammende Frage *B*. Diese ist mit ja zu beantworten und zwar so: Man konstruiert für die Diskriminantenfläche die Gl. der so viel als möglich berührenden Kugeln von so viel Dimensionen. Dann teilt man den ganzen Raum in Cuben *C* $\frac{1}{2^n}$ Volumen jener Kugel d.h. endliche Anzahl Cuben, wo in jedem Cubus nur ein Punkt zu nehmen und auszuprobieren ist.” (Wissenschaftliches Tagebuch I, p. 50.; this entry is summarised in the prospectus of highlights inlaid in the first notebook as “Frage *B*, ob Frage *A* durch endl. Zahl von Operationen entscheidbar”)

58 A remark found towards the end of the third notebook indicates that Hilbert did not continue to hold such a rigid view about the priority of superordinate questions. He writes (on p. 98 of the third notebook): “Es giebt Probleme, bei denen die Oberfrage wahrscheinlich dadurch am leichtesten beantwortet wird, dass man das Problem selbst löst z.B. $p-q=2$ auf $\infty$ viele Arten zu lösen, wo *p*, *q* Primzahlen sind; aber auch umgekehrt z.B die $10^{(10^{10})}$te Ziffer in der Dezimalbruchentwicklung für $\pi$ angeben” (Wissenschaftliches Tagebuch III, p. 51ff.). Connecting this entry with the entries in the first notebook is Hilbert’s example, “a $C_6$ with 11 separate ovals”, which is still on Hilbert’s mind in the third notebook as “the question whether a surface of 4th order with 11 separate ovals exists”, of which Hilbert says there: “Probleme, wie die Frage, ob es eine Fläche $4^\text {ter}$ Ordnung mit 11 getrennten Ovalen giebt, oder welches die Maximalzahl der sich einander ausschliessenden Züge einer ebenen | Curve 8ter Ordnung ist, sind von der Art, dass die zugehörige Oberfrage d.h. die Frage, ob jenes Problem durch endliche Anzahl von Schritten entscheidbar ist, zu bejahen ist, aber diese Probleme sind von derselben Art, wie das Problem die $10^{\left( 10^{10}\right) }$ Ziffer in der Dezimalbruchentwicklung von $\pi ^\pi$ zu finden.” (Wissenschaftliches Tagebuch III, p. 87f.)

59 “Unter 100 Gedanken, die zunächst an sich gleich gut sind, hat vielleicht einer durchschlagenden Erfolg. Man hat letzteren aber nicht von vorne herein in der Gewalt. – Man muss eine Sache so lange durchdenken, bis man sich genau Rechenschaft ablegen kann, warum man nicht weiter kommt, d.h. welche vorläufig zu complicirten aber durchführbaren Vorarbeiten geleistet werden müssen, um die gestellte Aufgabe zu lösen d.h. man muss wenigstens die nächste Oberfrage $B=F^2$ lösen.” (Wissenschaftliches Tagebuch I, p. 51)

60 “Häufig, wenn man durchaus ein Problem nicht lösen kann, liegt das daran, dass man ein anderes näher liegendes noch nicht gelöst hat: Wenn man sich die Strümpfe und dann die Stiefel ausziehen will wird das unmöglich, umgekehrt dagegen geht es ganz leicht.” (Wissenschaftliches Tagebuch III, p. 117).

61 “Was die Theorie von Problemstellungen betrifft, so kann man in der Zahlentheorie leicht beliebig schwierige Probleme stellen, die gar keine Angriffspunkte bieten. Da muss man dann zunächst die nächst leichteren Probleme suchen.” (Wissenschaftliches Tagebuch III, p. 133).

62 The full report, which, by the standards of these reports, is rather extensive, reads: “*Sitzung vom 21. Dezember*: D. Hilbert teilt einige allgemeine Betrachtungen über mathematische Probleme mit. Er ordnet dabei jedem Problem *A* ein “Oberproblem” *B* zu, das in der Frage besteht, ob das Problem *A* sich durch endlich viele Schritte lösen läßt, und zeigt an einigen Beispielen, daß die Lösung des Oberproblems *B*, obwohl leichter als die des Problems *A*, keineswegs trivial zu sein braucht, ja sogar – wie beim Fermatschen Satz – sehr schwierig sein kann. – Im Anschluß hieran erwähnt er die nun im Druck vorliegenden Vorträge, die Hr. Poincaré im April in Göttingen gehalten, und erörtert insbesondere die dort gegebenen Ausführungen zur Mengenlehre.” (Gutzmer 1910, p. 28 of the *Mitteilungen und Nachrichten*). This report shows how the assertion made by Thiele (2003, p. 8) that after 1904 “a thirteen-year break from the circle of ideas under discussion here ensued” must be qualified; also in other respects the claim that Hilbert ever took a total break from foundational reflection seems dubious (e.g. on account of Cod. Ms. D. Hilbert 559, see footnote 15, which I discuss further in an article tentatively entitled “Hilbert at Work between 1905–1917”).

63 Hilbert (1935, p. 310).

64 Hilbert (1918, p. 153).

65 That Hilbert appears to be exhuming the heart of his long buried sketch for a $24\text {th}$ Problem – apparently without having any progress to report – on this occasion is worth noting because this observation clarifies the character of Hilbert’s 1917 lecture on “Axiomatisches Denken”, which is widely seen as marking the beginning of a new phase in Hilbert’s career (cf. the introductory remarks to the English translation in Ewald (2005, pp. 1088, 1105)). Insofar as this assessment of its status is true at all, it is so only in the very limited sense of resulting in Hilbert’s first *publication* on foundational issues since 1904. In any other sense, the assessment is misleading or even wrong: As the examination of unpublished lecture-notes and manuscripts like Hilbert’s notebooks reveals, Hilbert had never ceased *thinking* (in private) or lecturing (see footnote 15, sometimes even to colleagues, not only students, see footnote 61) on foundational issues; the comparison of the text of “Axiomatisches Denken” with these unpublished documents furthermore shows that only a fraction of the material presented there was new in 1917: Even what is often cited as the main methodological innovation of “Axiomatisches Denken”: that one can distinguish “strata of axioms” (“Axiomschichten”, see Hilbert (1918, p. 148)) located at varying depth, already appears in the third notebook, where Hilbert observes: “Man kann in jeder Wissenschaft verschiedene Axiomschichten unterscheiden, z.B. meine früheren Grundlagen der Geometrie, und die jetzige Rückkehr auf den Punkt als alleiniges Element. Der Physiker nimmt einfach alle Thatsachen als ein Axiomensystem hin, das ist die leidlich erste und gröbste Schicht; Jeder ist ein Arbeiter in Axiomen, nur in verschiedenen Schichten.” (Wissenschaftliches Tagebuch III, p. 39); Hilbert also already speaks, as in “Axiomatisches Denken”, of “deeper” and “higher” such strata in the third notebook: “Jede tiefere Axiomschicht bedeutet eine abstraktere philosophischere Erkenntniss, jede höhere Axiomschicht ein Fortschritt nach der positiven Seite hin – wie die Baumringe. Die einzelnen Axiomschichten sind zugleich die wahren Ruhepunkte u. Stationen. Die Axiomatische Methode ist die Methode der Zukunft. Meine Geometrie als Beispiel nehmen. Zähle auf, was dabei Alles zur Sprache kommt und wie es neu und übersichtlich geordnet erscheint. Meine Lebensaufgabe, die getrennt überlieferten Wissens|zweige vereinigen (innerlich durch die Axiomatische Methode, nicht blos äusserlich wie Klein).” (Wissenschaftliches Tagebuch III, pp. 113f.) (The dating of these entries is uncertain, as noted in footnote 6, but they certainly predate 1917 by several years, possibly by as much a decade). That the other metaphor often quoted from Hilbert (1918, p. 146): that (mathematical) theories are like a certain type of building (“Fachwerke”), is not new in Hilbert’s mind in 1917 has been clear since the publication of Hilbert’s early lectures on geometry, where it appears as early as 1894 (see: Hilbert 2003, p. 72); it is, in any case, not original to Hilbert: one finds, e.g. Herbart in §13 of the third edition of his *Lehrbuch zur Einführung in die Philosophie* (1834, Herbart 1891, p. 51) already warning against “sich gewisse Fachwerke einzuprägen”, meaning by “Fachwerke” pre-made (philosophical) theories (presumably Herbart has in mind, e.g. Hegel’s System) which facts are then, bend or break, made to fit). In this light, “Axiomatisches Denken” appears like the sum-total of Hilbert’s foundational reflection up until 1917, not in any sense a new beginning.

66 Wissenschaftliches Tagebuch I, p. 37; see footnote 38. In fact, Hilbert had raised a particular case of this general issue already on the first few pages of the notebook: “Beweisen, dass man durch eine endliche Anzahl von Operationen entscheiden kann, ob und wie viele ganzzahlige Lösungen $\phi (x', y,\dots )=0$ mit ganzzahligen Coefficien[ten] besitzt.” (Wissenschaftliches Tagebuch I, p. 7)

67 “Die Kroneckersche Forderung alles durch eine endliche Anzahl von Processen ausführen zu müssen, ist nicht klar z.B. *F*(*a*, *b*) sei $=0$ oder die kleinste von $ax^3+b$ dargestellte Primzahl. Dennoch ist es nicht möglich zu entscheiden, ob $ax^3+b$ eine Primzahl darstellt, obwohl nur *F*(*a*, *b*) Operationen dazu nothwendig wären. Dagegen wären wir völlig befriedigt, wenn etwa $F(a,b)<a!b!$ nachweisbar wäre. Vgl. meinen Beweis für die Endlichkeit der Invarianten.” (Wissenschaftliches Tagebuch III, p. 133)

68 “Über die Endlichkeit ausführbarer Operationen in de[r] Mathematik. Die Kronecker’sche Ansicht, dass der Nachweis dieser Endlichkeit dem Begriff erst eine sichere Grundlage gibt ist nicht logisch. Eher könnte man sagen, dass der Nachweis nothwendig ist, dass etwas unter den Begriff fällt. —” (Wissenschaftliches Tagebuch II, p. 96)

69 Kronecker (1901, p. VI).

70 Marion (1995, p. 193) has similarly emphasised that Kronecker probably did not regard “finitude” (in whatever sense) as *necessary* for the admissibility of concepts, as Hilbert seems to imply here. Rather, Kronecker held that « finitary »concepts are always *preferable*, but that concepts with no « finitary »definitions were also admissible, if “only as a last resort” (as the late Harrold Edwards put it when making the same point in Edwards (1988, p. 144). von Plato (2017) has also emphasised this point more recently with a reference to the same passage of Hensel’s introduction as I just referred to.

71 When Hilbert visited Berlin in 1888 (as documented in Hilbert’s travel diary: Cod. Ms. D. Hilbert 741:1, see footnote 22). In a draft letter dated 11/XII/1888 (Cod. Ms. D. Hilbert 457:21 Hilbert thanks an unnamed “Hochgeehrter Herr Professor” for his “gütige Entgegenkommen” during Hilbert’s last visit to Berlin; that the intended recipient is Kronecker is likely already because of the reference to Berlin, but almost certain in light of Hilbert’s reference in the letter to the “von Ihnen begründete [...] The[orie] der Modulz[ahlen]”.

72 Hilbert expressly refers to what “he [Kronecker] expressed to me in person” on Hilbert (2013).

73 Cod. Ms. D Hilbert 741:1, fol. $2^r$ (see footnote 22). Rowe remarks that Kronecker’s remarks about invariant-theory must have felt for Hilbert “wie eine kalte Dusche” (see: Rowe 1995, pp. 545 sq.).

74 (Wissenschaftliches Tagebuch I, pp. 54.) (Rowe 1989, p. 196) reports another instance where “Hilbert’s self-confidence and plucky character” revealed itself when Hilbert defended the merits of his own work, in the case detailed by Rowe: against the criticisms Paul Gordan made of Hilbert’s early work in invariant theory.

75 Under the signature Cod. Ms. D. Hilbert 197 two letters and a postcard from Kronecker to Hilbert are found; in addition, three drafts of letters Hilbert wrote to Kronecker survive under the signatures Cod. Ms. D. Hilbert 457, items 9, 11 & 21. The letters Hilbert actually sent to Kronecker have, as far as I am aware, not survived.

76 See Hilbert’s draft letter to Kronecker dated 7/III/1890, i.e. Cod. Ms. D. Hilbert 457, item 9.

77 “ein ausserordentlich unbegreifliches Versehen” (see the letter dated 4/VII/1890 in the folder containing what survived amongst Hilbert’s papers of his correspondence with Kronecker: Cod. Ms. D. Hilbert 197)

78 In the third notebook and so (more than) a decade after Kronecker’s death in 1891, Hilbert still writes: “Von Kroneckers allgemeiner Anschauung kann man wohl sagen, dass die Wahrheit, die ja meist in der Mitte liegen soll, hier ganz auf einer Seite nämlich auf der Gegenseite zu Kronecker liegt.” (Wissenschaftliches Tagebuch III, p. 120)

79 This remark is found on a loose sheet kept as part of the convolute held by the SUB Göttingen under the signature “Cod. Ms. D. Hilbert 601”: “Sprache allein ohne Experiment ist unverständlich. Also Kroneckersche Entscheidbarkeit muss hinzukommen.”. The sheet in question has been numbered “31/32” by an archivist; the double-number is due to the fact that the sheet has been folded (like a greeting card), thus has two recto-sides.

80 The formulations flanking the sentences quoted in footnote 79 most closely resemble Hilbert’s discussion of Richard’s paradox in (the preparatory notes for) his lecture-course in 1914/15, as I will discuss in more detail in an article I am preparing under the title “Hilbert, Poincaré, Russell and Richard’s Paradox”.

81 As Poincaré also emphasised, insisting “on ne peut [expérimenter] que sur des objets matériels” (Poincaré 1891, p. 773).

82 “Die Punkte, Geraden, Ebenen in meiner Geometrie werden nie etwas anderes als Gedankendinge und haben als solche nie etwas mit wirklichen Punkten, Geraden, Ebenen zu thun. Ganz anders ist es mit meinen Dingen “oder”, “und”, “nicht”. Es ist eine Pikanterie, die einzig in der Wissenschaft dasteht und diesen Untersuchungen über das Denken einen eigenen Reiz giebt, dass da plötzlich die “und”, “nicht”, “oder” auch die Bedeutung wirklichen “oder”, “nicht” “und” erlangen, indem wir mit diesen Dingen auf den Papier gewissermassen experimentiren, während wir mit den Punkten, Geraden, Ebenen, niemals experimentiren durften. Es ist da die Region, wo die Grenze zwischen grob sinnlicher Empirie und reinstem Denken in einanderfliessen und man als Logiker zugleich das thut, was in der Geometrie der Geodät, in der Elektrizitätstheorie der Experimentator thut, nämlich mit den Dingen “oder”, “und”, “nicht” auf dem Papier experimentiren.” (Wissenschaftliches Tagebuch III, p. 101)

83 See: Hilbert and Bernays (1934, p. 32). I want to underscore that, even though these ideas appeared in print for the first time in the exposition of the finitst standpoint in *Grundlagen* I, Hilbert had been thinking about these matters, as the entry from the third notebook I just quoted shows, in more or less similar terms (of “experimentation” with “objects of thought”), since the early 1910s at least.

84 Hilbert and Bernays (1934, p. 42). Because Bernays, as Hilbert acknowledges in his Preface, effectively wrote the *Grundlagen der Mathematik* entirely by himself, it remains somewhat uncertain whether such minor historical remarks reflect Hilbert’s own judgement or only that of Bernays (which, in any case, should be considered more neutral, because Bernays was too young to have (negative) personal feelings about Kronecker); still, even if this tribute to Kronecker as a pioneer of the finitist standpoint is only Bernays’ opinion, the remark is nonetheless significant since it shows that Hilbert’s closest collaborator, Bernays, saw a clear affinity between Hilbert’s later methodological standpoint and Kronecker’s.

85 Hilbert and Bernays (1934, p. 32).

86 Lolli (2001, p. 207) has pointed out that one important effect of Gödel’s incompleteness proof was to stress “the important conceptual distinction between the decidable and the semi-decidable”. Whilst I think the entry I have just discussed shows that Hilbert sensed some such distinction between a wider and a narrower sense of “decidable” long before, it also shows that Hilbert did not succeed in conceptualising it distinctly.

87 See Behmann (1922, pp. 166 sq.). The Jahresberichte der Deutschen Mathematiker-Vereinigung (vol. 30, 1921) record, in the “Mitteilungen und Nachrichten”-section, p. 47, that Behmann gave a presentation with the title “Das Entscheidungsproblem der mathematischen Logik” to the mathematical colloquium in Göttingen on 10/5/1921. The text of this presentation has been published and translated by Mancosu and Zach (2015). Amongst Behmann’s papers there remains unpublished the manuscript of an entire (semester-long) lecture-course on the decision problem, which Behmann taught in the Wintersemester 1923/24 (the manuscript is item 45 of Kasten 9 of the Behmann-Nachlass held by the Staatsbibliothek Preußischer Kulturbesitz in Berlin as “Nachlass 335 (Behmann)”; it comprises 61 pages, with one side filled with Behmann’s miniscule handwriting and the other side mostly left blank or used for notes or calculations that are not part of the lecture-notes proper), wherein the problem is formulated in general terms on p. 5; a preliminary draft of these lecture-notes survives, furthermore, in an exercise-book Behmann apparently used to prepare lectures and presentations prior to writing out clean notes for himself. This draft is found in the item labelled “Heft 44”, bearing the title “Vektor und Tensorrechnung WS38/39”, in Kasten 6, on fols. $32^r$–$48^r$. Following this draft on fols. $48^v$–$49^r$ is another the draft of another presentation about the Entscheidungsproblem (yet another draft of the same text is found on a separate sheet inlaid in front of the exercise-book 44), different from that published by Mancosu & Zach: Behmann gave this presentation (as a set of more polished notes of this presentation found in Kasten 7 indicates) in Marburg in 1923; in attendance was, amongst (presumably) others, also Brouwer, of whose contribution to the discussion Behmann made a note (on p. 2 of the version in Kasten 7; fol. $49^r$ in Heft 44 of Kasten 6), which recalls that Brouwer expressed doubts about the (uncritical and indiscriminate) use of “and so on” in mathematical language.

88 This is how Behmann himself motivates his problem in the lecture published by Mancosu (2015, p. 178): Behmann contrasts the development of uniform methods for dealing with certain classes of calculation-problems with mathematical proof where still “*a new ingenious thought is needed in each case*” (Mancosu & Zach’s translation “creative” for “erfinderisch” here strikes me as unhappy, especially after they translated “erfinderisch” with “inventive” in the preceding paragraph (and again later when it occurs on p. 6 of Behmann’s manuscript), because Behmann also uses the word “schaffend” as an adjective corresponding to the word “Schöpfung”, which carries philosophically contentious implications, and Zach & Mancosu translate “schaffend” (appropriately) as “creative”).

89 Hilbert (2013, p. 608).

90 The official lecture notes read: “Wie bereits gesagt, ist für den allgemeineren Fall, dass die logischen Funktionszeichen auch zwei oder mehrere Argumente haben können, die Entscheidungsmöglichkeit bisher nicht erwiesen. Die Lösung des Entscheidungsproblems ist für die Fragen der Axiomatik von grundsätzlicher Wichtigkeit. [...] Wir wollen uns klar machen, dass mit der Lösung des Entscheidungsproblems ein Verfahren gegeben wäre, durch das jene Unbeweisbarkeit sich wenigstens grundsätzlich feststellen lassen müsste, (wenn auch vielleicht die Umständlichkeit des Verfahrens die praktische Durchführung illusorisch machen könnte.)” (Hilbert 2013, p. 542). In the corresponding place in Kneser’s notes, one reads: “Um aber weiter zu kommen, brauchen wir mindestens eine Relation, die der Identität $\equiv (x, y)$. Auch mit dieser ist das Entscheidungsproblem zu lösen. Herr Behmann hat es auch noch etwas weiter getrieben. Die allgemeine Lösung, auch bei nur zwei Relationen mit zwei Argumenten, stellt ein äußerst schwieriges mathematisches Problem vor.” Hilbert (2013, p. 609).

91 For example, Sieg (2013, p. 111) claims that “[c]learly, the general problem of mechanically deciding mathematical questions had been mentioned already [before the winter term of 1922/23]”; see also footnote 100.

92 Mancosu et al. (2010, p. 62) maintain, for example, that “[t]he origin of the decision-problem in Hilbert’s work is no doubt his conviction [...] that every mathematical problem has a solution”.

93 Re-cast in the wake of the development of strictly regimented artificial languages, the question whether computations involve finitely many operations turns into the question whether a given « way of obtaining a result »– as most will have been taught to think of it: an operation on the domain of a model suitable for the language – can be defined by a (finitely long) function-term in the language.

94 That the term “effective” should be given the precise sense “general recursive” has become known as the “Church-Turing Thesis”. Hilbert, however, at least in some of the passages I have been discussing, seems to have had in mind primarily primitive recursive functions (see the discussion on p. 27). Tait (2005, p. 29) also prominently advocated primitive recursive arithmetic as a rational reconstruction of Hilbertian finitism. To the historian Hilbert’s conception of finitism presents something of a moving target: In lectures in February 1922 Hilbert introduced a kind of minimisation-operator (see: Hilbert 2013, pp. 589 sq.), the acceptance of which marks the step from primitive to general recursive operations as now standardly defined. Zach (2003, p. 235) has noted, furthermore, that Hilbert’s endorsement of Ackermann’s work indicates that Hilbert found (certain forms of) transfinite recursion finitistically acceptable; indeed, in Hilbert (1930a, pp. 293, 307 sq.) (which Zach does not discuss), Hilbert appears to countenance transfinite induction explicitly.

95 The “curious” fact (noted by the editors in their introduction to: Hilbert 2013, p. 42) “that Hilbert does not mention the decision problem in the 1917/18 lectures” is evidence that Hilbert did not arrive at the idea of Behmannian Decidability on his own even after he had in hand what can, with the added wisdom of retrospect, be recognised as its two key components: (a) precisely defined deductive systems in strictly regimented artificial languages (which Hilbert first developed in 1917/18) and (b) his own normal-form-result from 1905 which is an example of a mechanical decision procedure for the correctness of certain formulas.

96 In particular, in §49, which is entitled “Kurze Besprechung zweier Fragen, die wesentlich axiomatischer Natur sind”. Behmann writes there: “Es sind namentlich in neuerer Zeit gewisse mathematische Fragen hervorgetreten, bei denen wir nicht nur nicht wissen, welchen Weg wir zu ihrer Entscheidung zu wählen haben, sondern nicht einmal dies, ob sie aufgrund der uns bekannten Axiome überhaupt entschieden werden können, und zu denen wir daher schwer eine befriedigenden Standpunkt gewinnen können. Und zwar handelt es sich hier namentlich um Fragen wie diese, ob, wenn irgend eine bestimmte reelle Zahl wie z.B. $e^\pi$ vorgelegt ist, es notwendig entscheidbar ist, ob sie rational oder irrational bez. algebraisch oder transzendent ist. (Ganz von selbst gelangen wir von hier aus auf die Frage, ob das zugrunde liegende Axiomensystem die Eigenschaft der Vollständigkeit hat, d.h. ob jede vernünftig gestellte Frage einer Beantwortung in dem gemeinten Sinne fähig ist.)” (Behmann 1918, p. 324). He goes on to point to Cantor’s continuum-problem as an example of such a question and remarks: “Solange aber weder dies Letzte [i.e. that the existence of a function mapping real numbers to a certain section of ordinal numbers] bewiesen noch eine passende Funktion aufgezeigt werden kann, muss die Frage natürlich als unentschieden gelten. Vielleicht ist es sogar möglich, die Unentscheidbarkeit ihrerseits zu beweisen oder gar die Unentscheidbarkeit der Entscheidbarkeitsfrage. Hinsichtlich der grundsätzlichen Möglichkeit derartiger Fälle Klarheit zu schaffen, erkennt man schon allein hieraus als eine dringende Aufgabe.” (Behmann 1918, p. 325; Mancosu 2010, p. 196) likewise sees in these particular passages from Behmann’s dissertation “a statement of what will become the general ‘Entscheidungsproblem’”.

97 In a letter from Behmann to Heinrich Scholz (dated 27/XII/1927 and kept along with Behmann’s other correspondence with Scholz in box 3 of the Behmann Papers at the SBB Berlin). After mentioning Hilbert’s decision procedure for sentential logic, Behmann says that nobody raised the general decision-problem before him.

98 That Behmann is aware not just of Hilbert’s published work, but also of his recent lecture-courses on logic is attested by a reference to Hilbert’s 1917/18 lecture course in a footnote (Behmann 1922, p. 165, footnote 2). In the text published recently by Mancous & Zach, Behmann makes reference to Hilbert’s 1900 address (see: Mancosu and Zach 2015, p. 179), but only to support the general methodological point that any problem can be settled positively *or negatively*. Indeed, Behmann’s description of his project, as aiming at “a bracketing of thinking in favour of mechanical calculation” (Mancosu and Zach 2015, p. 180), places him in some opposition to Hilbert’s attitude as expressed, e.g. in the passage quoted in footnote 51.

99 Behmann emphasises the value of the algebra of logic as precedent in general terms in the lecture published by Mancosu and Zach (2015, p. 180 sq.). In the historical account of the discovery of his decision-problem given in Behmann’s first letter to Russell (dated 8/8/1922, accompanying an offprint of Behmann’s 1922 article) Behmann stresses, in particular, the importance of what Schröder in his *Vorlesungen über die Algebra der Logik. Exakte Logik* called the “Eliminationsproblem” (Schröder 1890) (Behmann mentions Schröder’s work, and the elimination-problem in particular, also in his letter to Scholz referred to in footnote 97. On p. 5 of the lecture-notes mentioned in footnote 87, Behmann goes so far as to proclaim his work the “heir” to the algebraic tradition in logic. Since the most plausible prefiguration of Behmann’s Entscheidungsproblem in Hilbert’s work, Hilbert’s normal-form-result for sentential logic in 1905 (see footnote 95), was likewise derived from Schröder (as I have documented elsewhere), the one person, who may plausibly be said to have, however distantly, grasped the idea of Behmannian Decidability and the Entscheidungsproblem before Behmann was Ernst Schröder.

100 Rowe (2000, p. 85) speaks of an “underlying tension between transcendental and constructive arguments”, which also describes well the issue I think Hilbert’s conviction of the solvability of all problems leaves open. (On p. 84 Rowe also claims, however, that Hilbert had in 1917 started “thinking about the so-called *Entscheidungsproblem*”.)

101 “Die Eigenschaften des Denkens 1.) streng und korrekt ist nur das, was wie experimentell $2+1=1+2$ etc sicher ist. 2.) Dass jeder math. Satz beweisbar ist, hängen gewiss tiefer zusammen und entspringen aus einer gemeinsamen Quelle.” (Wissenschaftliches Tagebuch III, p. 93)

102 Hilbert’s talk of a “common source” may (consciously or not) be echoing Kant’s talk of “Grundquellen” from which all knowledge flows (Kant 1998, A50/B74); or of Kant’s cryptic allusion to a “gemeinschaftliche, aber uns unbekannte Wurzel” of both intuition and cognition (Kant 1998, A15/B29).

103 Thiele (2003, p. 7) speculates, in my opinion not implausibly, that Hilbert chose not to publicise his $24\text {th}$ Problem in 1900 because he had not managed to make enough progress in addressing it to be confident that its pursuit would be worth while; (see also: Thiele and Wos 2002, pp. 73, 87).

104 All translations into English are mine. Where digitisations of unpublished sources are freely available, but not in readable editions, transcriptions of the relevant passages are provided in footnotes. Verbal quotations are flanked by the usual English quotation marks; French guillemets serve as typographically distinct « scare-quotes »on occasion.
